# Ezrin Is Associated with Disease Progression in Ovarian Carcinoma

**DOI:** 10.1371/journal.pone.0162502

**Published:** 2016-09-13

**Authors:** Vered Horwitz, Ben Davidson, Dganit Stern, Claes G. Tropé, Tali Tavor Re’em, Reuven Reich

**Affiliations:** 1 Institute of Drug Research, School of Pharmacy, Faculty of Medicine, The Hebrew University of Jerusalem, Jerusalem, Israel; 2 Department of Pharmacology, Israel Institute for Biological Research, Ness Ziona, Israel; 3 Departments of Pathology, Oslo University Hospital, Norwegian Radium Hospital, Oslo, Norway; 4 University of Oslo, Faculty of Medicine, Institute of Clinical Medicine, Oslo, Norway; 5 Gynecologic Oncology Oslo University Hospital, Norwegian Radium Hospital, Oslo, Norway; 6 Department of Pharmaceutical Engineering, Azrieli College of Engineering, Jerusalem, Israel; 7 David R. Bloom Center for Pharmacy at The Hebrew University of Jerusalem, Jerusalem, Israel; 8 Adolf and Klara Brettler Center for Research in Molecular Pharmacology and Therapeutics at The Hebrew University of Jerusalem, Jerusalem, Israel; The University of Hong Kong, HONG KONG

## Abstract

**Objective:**

Ezrin and p130Cas are structural proteins with an important role in signaling pathways and have been shown to promote cancer dissemination. We previously reported on overexpression of both ezrin and p130Cas in breast carcinoma effusions compared to primary carcinomas. Since ovarian and breast carcinomas share the ability to disseminate by forming malignant effusions, we sought to study the role of these molecules in ovarian carcinoma (OC).

**Methods:**

OC cell lines were cultured in two different 3-dimensional conditions, on alginate scaffolds and as spheroids, which served as models for solid tumor and malignant effusions, respectively. shRNA was used to reduce protein expression in the cells. The malignant potential was evaluated by chemo-invasion assay, branching capacity on Matrigel and rate of proliferation. Subsequently, clinical specimens of high-grade serous carcinoma effusions, ovarian tumors and solid metastases were analyzed for ezrin and p130Cas expression.

**Results:**

Higher ezrin expression was found in cells composing the spheroids compared to their counterparts cultured on alginate scaffold and in clinical samples of malignant effusions compared to solid tumors. In addition, reduced Ezrin expression impaired the invasion ability and the branching capacity of OC cells to a greater extent than reduced p130Cas expression. However, ezrin and p130Cas expression in effusions was unrelated to clinical outcome.

**Conclusions:**

The 3-dimensional cell cultures were found to mimic the different tumor sites and be applicable as a model. The *in vitro* results concur with the clinical specimen analysis, suggesting that in OC, the role of ezrin in disease progression is more pronounced than that of p130Cas.

## Introduction

Ezrin and p130Cas are two different cytosolic structural proteins that play an important role in signaling pathways affecting the cytoskeleton and regulating cell motility and proliferation [1,2].

Ezrin is part of the ERM (ezrin, radixin, moesin) family of proteins that function as linkers between the plasma membrane and the actin cytoskeleton. As such, they are placed at the center of a regulatory network of many cellular processes, in both physiological and pathological conditions [2,3]. Ezrin is a cytosolic non-phosphorylated protein in its dormant form. Following phosphorylation, the active ezrin (p-ezrin) translocate to the cell membrane and interacts with transmembrane proteins, as well as the cytoskeleton, regulating cell morphology and motility and transducing growth signals [2,4]. Ezrin or p-ezrin overexpression has been found in diverse human cancers and has been associated with tumor progression and malignant phenotype [5–12]. Ezrin has been shown to promote cancer dissemination by several mechanisms including changes in signaling, increased cell motility and the ability to survive anoikis, invade and proliferate in 3-dimensional environment [2,7,10,13].

p130Cas is an adaptor protein that functions as an important signaling node in many signaling pathways in the cell and acts as an important regulator in cytoskeleton organization and cell motility and survival. p130Cas coordinates different signaling pathways, including growth factor receptor tyrosine kinases, non-receptor tyrosine kinases, integrins and mechanical signaling [1,14,15]. Phosphorylation regulates p130Cas, mainly in tyrosine residues located in the substrate domain of the protein, resulting in protein translocation to the cell membrane and association with adhesion proteins [1,14]. p130Cas overexpression and hyper-phosphorylation were shown to be associated with malignant phenotype in several cancers [16–20].

Ovarian cancer, consisting predominantly of ovarian carcinoma (OC), is the fifth most common cancer in the United States and the leading cause of death from gynecological cancer in Western countries [21]. Only 25% of OC are diagnosed at stage I, and it is associated more than any other carcinoma with the accumulation of malignant effusions in the peritoneal cavity (ascites) [22]. Studies of ovarian solid tumors showed that higher ezrin or p130Cas have been associated with advanced stage and poor prognosis [23–25]. However, no data is available on these proteins in OC effusions.

We previously compared the gene expression profiles of primary breast carcinomas and breast carcinoma effusions, and found ezrin and p130Cas to be upregulated in effusion specimens at the mRNA and protein level [20]. In a subsequent *in vitro* study, both ezrin and p130Cas mediated the growth of anchorage-independent breast carcinoma cells as 3-dimensional spheroids that were used as a model for breast carcinoma metastasis [12].

In contrast to the solid primary tumors or solid metastases, tumor cells in effusions are suspended as clusters. Ovarian and breast carcinomas share the ability to disseminate by forming malignant effusions, and we therefore sought to compare the role of ezrin and p130cas in these tumors.

In the present study, the roles of ezrin and p130Cas in OC were studied *in vitro* in OC cell lines cultured in two different 3-dimensional conditions, on alginate scaffolds and as spheroids, which served as models for solid tumor and malignant effusions, respectively. Ezrin and p130Cas expression was additionally measured in clinical specimens of OC effusions, the ovarian tumors and solid metastases.

## Materials and methods

### Cell lines

ES2 (CRL-1978) and OVCAR3 (HTB-161) OC cells were obtained from the American Type Culture Collection (ATCC) (Manassas, VA). Cells were maintained in DMEM supplemented with 10% fetal calf serum, penicillin, streptomycin, amphotericin, L-glutamine, sodium pyruvate, vitamins and non-essential amino acids (Biological Industries, Beit Haemek, Israel). Cell cultures were incubated at 37°C in humidified atmosphere of 95% air and 5% CO_2_.

### Patients and specimens

The material analyzed using real-time PCR consisted of 93 effusions (76 peritoneal, 17 pleural) from 93 patients diagnosed with high-grade serous carcinoma (HGSC). In view of the fact that the fallopian tubes have not been adequately assessed in this cohort, tumors localized to the ovary are referred to as ‘ovarian’, but not as primary. Specimens were submitted for routine diagnostic purposes to the Department of Pathology at the Norwegian Radium Hospital during the period of 1998–2008. OC specimens and clinical data were obtained from the Department of Gynecologic Oncology, Norwegian Radium Hospital. Clinicopathologic data are detailed in Table 1. The majority of patients (91/93; 98%) received platinum-based chemotherapy.

**Table 1:** Clinicopathologic parameters of the study cohort (93 patients).

Parameter	Number of patients
**Age (mean)**	38–83 years (62)
**FIGO stage**	
II	2
III	49
IV	42
**Residual disease**	
≤1 cm	43
>1 cm	35
NA ^*a*^	15
**CA 125 at diagnosis (range; median)**	11–43800 (982) ^*b*^
**Chemoresponse after primary treatment**	
CR	46
PR	24
SD	8
PD	9
NA ^*c*^	6

Abbreviations: NA = non available; CR = complete response; PR = partial response; SD = stable disease; PD = progressive disease

^*a*^ Including effusions from inoperable patients, patients who received neoadjuvant chemotherapy and patients with no record.

^*b*^ Available for 69 patients

^*c*^ Disease response after chemotherapy could not be evaluated because of normalized CA 125 after primary surgery or missing CA 125 information and no residual tumor.

All effusions were centrifuged immediately after tapping, and cell pellets were frozen at -70°C in equal amounts of RPMI 1640 medium (GIBCO-Invitrogen, Carlsbad CA) containing 50% fetal calf serum (PAA Laboratories GmbH, Pasching, Austria) and 20% dimethylsulfoxide (Merck KGaA, Darmstadt, Germany). Effusions were diagnosed by an experienced cytopathologist (BD) using morphology and immunohistochemistry (IHC), based on evaluation of smears and cell blocks prepared using the Thrombin clot method.

Fifty-seven solid HGSC specimens (33 ovarian, 24 peritoneal metastases, the majority omental) were studied. Frozen sections from all tumors were reviewed by a gynecopathologist (BD), and only specimens with tumor cell population >50% and minimal or no necrosis were included in this study.

Informed consent was obtained according to national and institutional guidelines. The Regional Committee for Medical Ethics in Norway approved the study (Ethics approval S-04300) and granted dispensation from obtaining informed consent for all patients whose specimens were submitted to the department of pathology at the Norwegian Radium Hospital in the years 1998–2006, since the majority of patients were deceased at the time of application. For patients with samples submitted from 2007 onwards, a written consent was obtained.

### RNA and protein isolation

Solid specimens were washed with PBS and then 500μl of Tri-reagent (Sigma-Aldrich, St. Louis MO) or RIPA buffer (nonidet P-40 1%, Tris HCl pH 7.5 20mM, NaCl 137mM, EDTA 0.5mM, glycerol 10%, protease inhibitor cocktail 1%, sodium orthovanadate 1mM and SDS 0.1% (Sigma)) were added for future RNA or protein extraction, respectively. Effusions were centrifuged (1000rpm, 7 min), washed with PBS, and lysed with 500μl Tri-reagent or 300μl RIPA buffer. Total RNA was extracted according to Tri-Reagent manufacture's protocol. Specimens in RIPA buffer were incubated for 30 min at 4°C and then centrifuged (14,000 rpm, 15 min, 4°C). The supernatant was used for Western blotting.

### Real-time PCR

Total RNA (1μg) was reverse-transcribed using qScript^TM^ cDNA synthesis Kit (Quanta Biosciences, Gaithersburg, MD). The cDNA sample, diluted 1:128, was further processed by real time PCR (CFX Connect^TM^ Real-Time System, Bio-Rad, Hercules CA), using KAPA SYBER FAST Universal qPCR Kit (Kapabiosystem, Boston MA). Primer sequences were as follows:

Ezrin: sense: 5'-CGCTCTAAGGTTCTGCTCT-3'

antisense 5'-TCCTGGGCAGACACCTTCTTA-3'

p130Cas: sense: 5'-GGGCCACAGGACATCTATGAT-3'

antisense: 5'-GAGGACGTCGTAGACTGCG-3'

Radixin: sense: 5'-TTTGAAGCAATGTGGGGACC-3'

antisense: 5'-AGGGCCCTGGGTAATGGAAT-3'

RPLP0 (reference gene): sense 5'-CCAACTACTTCCTTAAGATCATCCAACTA-3'

antisense 5'-ACATGCGGATCTGCTGCA-3'

The Ct value for the reference gene was subtracted from the Ct value, generating a ΔCt value, which was used for statistical analysis. ΔΔCt was calculated by subtracting the mean value of ΔCt of the HGSC from each sample.

### Western blotting

One-hundred and ten of the 150 specimens analyzed using Real-time PCR (65 effusions, 27 ovarian tumors, 18 solid metastases) were analyzed for Ezrin and p130cas protein expression by Western blotting. Total protein (25μg) was separated by electrophoresis on SDS-10% polyacrylamide gels under reducing conditions and transferred to Immobilon transfer membranes (Millipore, Bedford, MA). Membranes were blocked in TBST (10mM Tris-HCl (pH 8.0), 150mM NaCl and 0.1% Tween 20) containing 5% DifcoTM skim milk (BD, Sparks, MD) for 1h at room temperature, and then incubated overnight in 4°C in 5% BSA in TBST containing either rabbit anti-phospho-ezrin (Thr567)/Radixin (Thr564)/Moesin (Thr558) antibody (termed in this study as p-ERM), anti-phospho-p130Cas (Tyr165) antibody, anti-phospho-p130Cas (Tyr410) antibody, anti-p130Cas (E1L9G) antibody (Cell Signaling Technology, Danvers, MA) or anti-ezrin antibody (ab41672, Abcam, Cambridge, UK). Membranes were then washed with TBST followed by 1h incubation with Peroxidase-conjugated AffiniPure Goat anti- Rabbit IgG (Jackson ImmunoResearch, West Grove PA) in TBST containing 5% skim milk. After being washed with TBST, membranes were imaged by enhanced chemiluminescence (ECL) (Pierce, Rockford IL), according to the manufacturer’s specifications using Image Lab 5.0 gel reader (Bio-Rad, Hercules CA). Membranes were washed with TBST and then incubated for 1h in 5% BSA in TBST containing rabbit anti-GAPDH (14C10) antibody (Cell Signaling Technology). Membranes were then processed as mentioned above. Protein lysate from NIH-3T3 cells served as control for the WB analysis. Densitometer analysis was performed using the Image J program.

### Tri-dimensional cell culture on alginate scaffold

Cells growth on alginate scaffold was used as a 3-dimensional model for solid tumor. Alginate scaffolds (diameter 5mm, thickness 2mm), were generated from LVG alginate (LVG with M.W. e 100 kDa, >65% guluronic acid monomer content, NovaMatrix FMC Biopolymers, Drammen, Norway). The macro-porous structure was obtained by a freeze-drying technique. For scaffold preparation, 600mg of the LVG alginate powder was added to the 50ml of sterilized double-distilled water (DDW; 1.2% (w/v) solution). Following 2h stirring on hot plate for complete dissolution, crosslinking was performed by calcium gluconate (1.2%w/v) using homogenizer apparatus. Final composition of cross-linked solution was 1% alginate and 0.2% (w/v) calcium gluconate. After an additional 30min of stirring, 100μl portions of the cross-linked solution were placed into 96-well NUNC plates. The plates were placed for 1h at 4°C and then frozen overnight at -20°C. The process was finalized by lyophilization, to obtain the macro-porous structure of the scaffold, and 45 minutes of UV sterilization.

Cells (400,000 in 15μl) were plated on the scaffold placed in the 96-well plate. Following centrifugation (750rpm, 3 min) and 30 min incubation (37°C), 50μl of supplemented DMEM were gently added to the scaffold. Plates were incubated for 30 min at 37°C, and scaffolds were then gently transferred to a 24-well plate containing 1.5ml supplemented DMEM and maintained for 1 (ES2) or 2 (OVCAR3) weeks. Cell lysis was performed by incubating 6 scaffolds in 100μl RIPA buffer for 30 min at 4°C followed by centrifugation (20,000g, 15 min, 4°C). The supernatant was used for Western Blotting analysis.

### Tri-dimensional cell culture as spheroids

Cell growth as spheroids was used as a model for malignant effusions. Cells (400,000 in 3ml supplemented medium) were added to 6-well plates placed on a rocker. The rocking prevented cell adhesion to the culture dish and resulted in spheroid-like cell growth. Cells that are cultured on non-adhesive matrix, such as sea agar, generate few small spheroid floating in the culture dish. Rocking culture results in multiple floating spheroids within the media, similarly to cell clusters seen in malignant effusions from patients.

Spheroids were photographed after 24h using an Olympus E-330 camera and Olympus CKX41 inverted microscope, 10X/0.25 PhP (Tokyo, Japan). The spheroids were counted and their diameters were measured in 5 random fields of the culture. On the following day, the spheroids were collected and centrifuged (1500rpm, 7 min), and 40μl RIPA buffer was added to the sediment. Following 30 min incubation at 4°C and centrifugation (20,000g, 15 min, 4°C), the supernatant was used for Western blotting analysis.

### Stable transfection with short hairpin RNA (shRNA)

MISSION^TM^ pLKO.1-Puro lentivirus plasmid containing five different selective shRNAs sequences against ezrin and p130Cas were obtained as Escherichia coli (E. coli) glycerol stocks from Sigma-Aldrich. shRNA sequences are detailed in Konstantinovsky et al. 2012 [12]. shRNA plasmid-containing bacteria were incubated overnight in freshly prepared Luria-Bertani (LB) medium supplemented with 100μg/ml ampicillin at 37°C in humidified atmosphere. Plasmid DNAs were purified from the E. coli culture using GeneJet^TM^ plasmid Miniprep kit (Fermentas Life Science, Vilnius, Lithuania), and plasmid concentration was measured by NanoDrop 2000 spectrophotometer system (Thermo Scientific, Waltham MA).

Based on results in the previous study [12], two plasmids were selected for ezrin and p130Cas each for transfection: for ezrin: clone c-5-3 TRCN0000062460 (T1) and clone c-3-1 TRCN0000062462 (T2) and for p130Cas clone c-5 TRCN000115982 (T3) and clone c-3 TRCN000115985 (T4) [12]. ES2 and OVCAR3 cells were plated in 24-well culture dish (100,000 cells per well). The following day, 700ng of purified plasmid were transfected using Lipofectamine® 2000 reagent (Invitrogen). Culture medium supplemented with 0.8μg/ml puromycin served as selective medium for successfully transfected cells and was used 2 days after transfection and thereafter. Cells that were transfected with no plasmid and non-transfected cells did not survive the puromycin treatment, and served as controls (C1 and C2, respectively).

### Proliferation assay

Cells were plated in a 6-well culture dish (50,000 cells per well). Viable cells were stained 24h later using 0.5mg/ml MTT for 15 min (Sigma). Cells were washed with PBS and then dissolved using 0.5ml DMSO. The absorbance of the solution was measured at 590nm using Multiskan RC plate reader (Thermo Scientific).

### Boyden chamber chemo-invasion assay

Matrigel (reconstituted basement membrane; 25μg) was dried on a polycarbonated filter (PVP free, Nuclepore, Whatman, Maidstone, UK). Fibroblast conditioned medium (obtained from confluent NIH-3T3 cells cultured in serum free DMEM) was used as the chemoattractant. Cells were harvested by brief exposure to 1mM EDTA, washed with DMEM containing 0.1% bovine serum albumin and added to the Boyden chamber (200,000 cells). Cells were incubated for 6h at 37°C in humidified atmosphere of 95% air and 5% CO_2_. Cells that traversed the Matrigel layer and attached to the lower surface of the filter were stained with Diff Quik kit (Dade Diagnostics, Aguada PR) and counted in five randomized fields. The mean of the counts was calculated for each cell line and are expressed as the mean ± SE.

### Branching capacity on Matrigel

Cells were harvested by brief exposure to 1mM EDTA, washed with DMEM containing 0.1% bovine serum albumin and plated on a 12-well plate covered with 250μg Matrigel (50,000 cells per well). Plates were incubated for 6h at 37°C in humidified atmosphere of 95% air and 5% CO_2_. OVCAR3 cells culture was monitored up to 24h. Cells were then photographed using an Axiocam 105 color microscope camera, magnification X10 (Zeiss, Oberkochen, Germany) and the number of junctions was counted.

### Statistical analysis

Statistical analysis was performed applying the SPSS-PC package (Version 21, Chicago IL). Probability of <0.05 was considered statistically significant. Comparative analysis of effusions, ovarian tumors and solid metastases were performed using the Kruskal-Wallis H test. Analysis of the association between mRNA and protein expression and clinicopathologic parameters for patients with effusion specimens was performed using the Mann-Whitney U test. For this analysis, as well as for survival analysis, clinicopathologic parameters were grouped as follows: age: ≤60 vs. >60 years; effusion site: peritoneal vs. pleural; FIGO stage: III vs. IV; chemotherapy status: pre- vs. post-chemotherapy specimens; residual disease (RD): ≤1 cm vs. >1 cm; response to chemotherapy: complete response vs. partial response/stable disease/progressive disease. The paired-sample T-test was used to analyze the association between mRNA and protein expression and serum CA-125 levels at diagnosis.

Progression-free survival (PFS) and overall survival (OS) were calculated from the date of the last chemotherapy treatment/diagnosis to the date of recurrence/death or last follow-up, respectively. Univariate survival analyses of PFS and OS were executed using the Kaplan-Meier method and log-rank test. Platinum resistance was defined as PFS≤6 months according to guidelines published by the Gynecologic Oncology Group (GOG) [26] and progressive disease or recurrence was evaluated by the RECIST criteria [27]. Multivariate survival analysis was performed using the Cox regression model (Enter function).

## Results

### OC cells cultured as spheroids and OC effusions have higher ezrin expression

**Fig 1:**
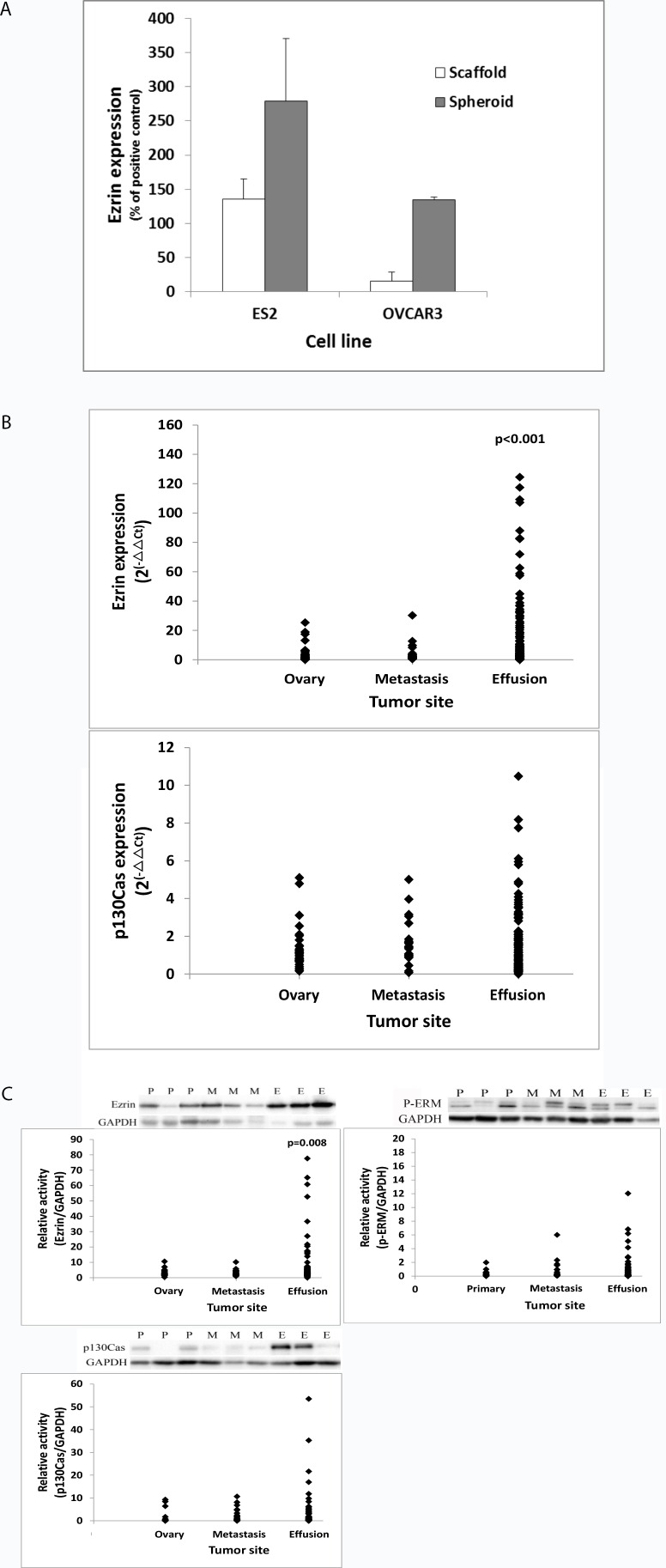
Expression of ezrin and p130Cas in cell lines and in clinical samples. (A) Two human OC cell lines, ES2 and OVCAR3, were cultured on alginate scaffolds and as spheroids, as models for solid tumors and malignant effusions, respectively. Ezrin protein level was higher in cells composing the spheroid compared to their counterparts on scaffolds, though not significantly. Sample distribution is presented for mRNA levels of ezrin and p130Cas (B) and protein levels of ezrin, p-ERM and p130Cas (C) at 3 different tumor sites: the ovary, solid metastases and malignant effusions. Representative blot is shown for the measured proteins and the corresponding reference protein (GAPDH) in ovarian tumors (P), metastases (M) and malignant effusions (E). Ezrin was significantly elevated at both the mRNA (B, p<0.001) and protein (C, p = 0.008) level in malignant effusions compared to the solid tumors. p130cas mRNA and protein and p-ERM protein levels were comparable at the 3 anatomic sites (B and C, p>0.05). Phosphorylated p130Cas was hardly detected, regardless of tumor site (not shown).

Analysis of ezrin protein expression in ES2 and OVCAR3 cells cultured on alginate scaffolds and as spheroids showed higher expression in the latter, though not significantly (Fig 1A). The effect of the culture condition on the expression of p130Cas was less pronounced (data not shown).

In agreement with this observation, in the clinical specimens, HGSC cells in effusions had significantly higher Ezrin mRNA (p<0.001; Fig 1B) and protein (p = 0.008; Fig 1C) levels compared to the solid lesions. p130cas mRNA and protein and p-ezrin levels were comparable at the 3 anatomic sites (p>0.05; Fig 1B and 1C) (S1 Fig). No radixin mRNA expression was detected in OC clinical samples, a human glioblastoma cell line, LN18 (generous gift of Professor Lazarovici, HUJI) was used as positive control.

Notably, two phosphorylated forms of p130Cas that were evaluated in this study, phospho-Tyr165 and phospho-Tyr410, were only weakly detected in few clinical samples, regardless of tumor site (data not shown).

### Ezrin and p130Cas are effectively silenced by shRNA

**Fig 2:**
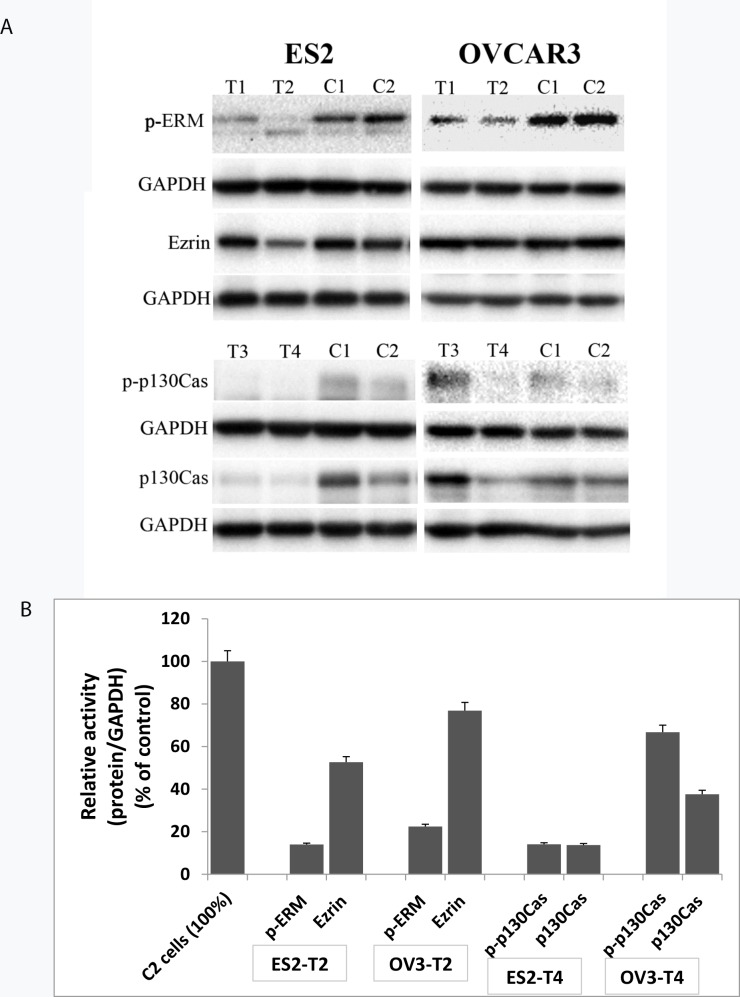
Reduced expression of ezrin and p130Cas following transfection with shRNA. ES2 and OVCAR3 ovarian carcinoma cells were transfected with shRNA containing plasmid. Reduced ezrin (T1, T2) or p130Cas (T3, T4) expression was verified by comparison with the expression in cells transfected with no plasmid (C1) and non-transfected cells (C2), all grown as spheroids. (A) For each cell line a representative blot is shown demonstrating expression of ezrin, p-ERM, p130Cas, p-p130Cas and the corresponding reference protein (GAPDH). ES2-T2 and OVCAR3-T2 clones were selected as cells with reduced ezrin expression and ES2-T4 and OVCAR3-T4 were selected as cells with reduced p130Cas expression for further studies. (B) Quantification of protein expression in the selected cell line is presented.

To further study the role of ezrin and p130Cas in OC, ES2 and OVCAR3 cells were transfected with shRNA-containing plasmids and the inhibition of expression was verified by Western Blotting analysis in comparison with the expression in cells transfected with no plasmid and non-transfected cells, all grown as spheroids (Fig 2). In OVCAR3-T2 cells, a reduction in both p-ERM and ezrin was seen, although the reduction is more evident in p-ERM. In ES2-T2 cells, a higher extent of the reduction in ezrin expression was seen compared to OVCAR3-T2 cells. ES2-T2 and OVCAR3-T2 clones were selected as cells with reduced ezrin expression and ES2-T4 and OVCAR3-T4 were selected as cells with reduced p130Cas expression for further studies. (For complete Western Blots see S2 Fig)

### Ezrin or p130Cas reduced expression does not affect spheroid morphology and proliferation rate

**Fig 3:**
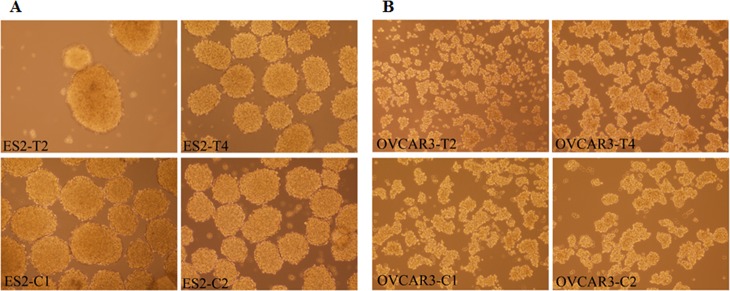
No change in spheroid morphology following reduction in ezrin or p130Cas expression. Following 24h incubation on rocking culture dish, (A) ES2 cells formed organized round-shaped spheroids that sometimes were fused to form large clusters: ES2-C1 and ES2-C2, cells transfected with no plasmid and non-transfected cells, respectively; (B) OVCAR3 cells formed less organized cell clusters that varied in shape and size: OVCAR3-C1 and OVCAR3-C2, cells transfected with no plasmid and non-transfected cells, respectively. The transfected cells formed clusters similarly to the control cells. Ezrin reduced expression: ES2-T2 in A and OVCAR3-T2 in B. p130Cas reduced expression: ES2-T4 in A, OVCAR3-T4 in B (Magnification X40).

Decreased expression of ezrin or p130Cas did not change the proliferation rate of ES2 and OVCAR3 cells, measured using the MTT viability assay (data not shown). When plated on rocking culture dish, both ES2 and OVCAR3 cells formed spheroid-like floating cell clusters. ES2-C1 and ES2-C2 cells formed organized round-shaped spheroids that sometimes were fused to form large clusters (Fig 3A). No significant change was found in the number of clusters nor in cluster size in ES2 transfected cells. OVCAR3-C1 and OVCAR3-C2 cells formed less organized cell clusters that varied in shape and size (Fig 3B). The transfected cells formed clusters similar to control cells, indicating that reduced expression of ezrin or p130Cas did not change significantly the morphology of the spheroids (Fig 3).

### Ezrin and p130cas affect cell branching capacity on Matrigel

**Fig 4:**
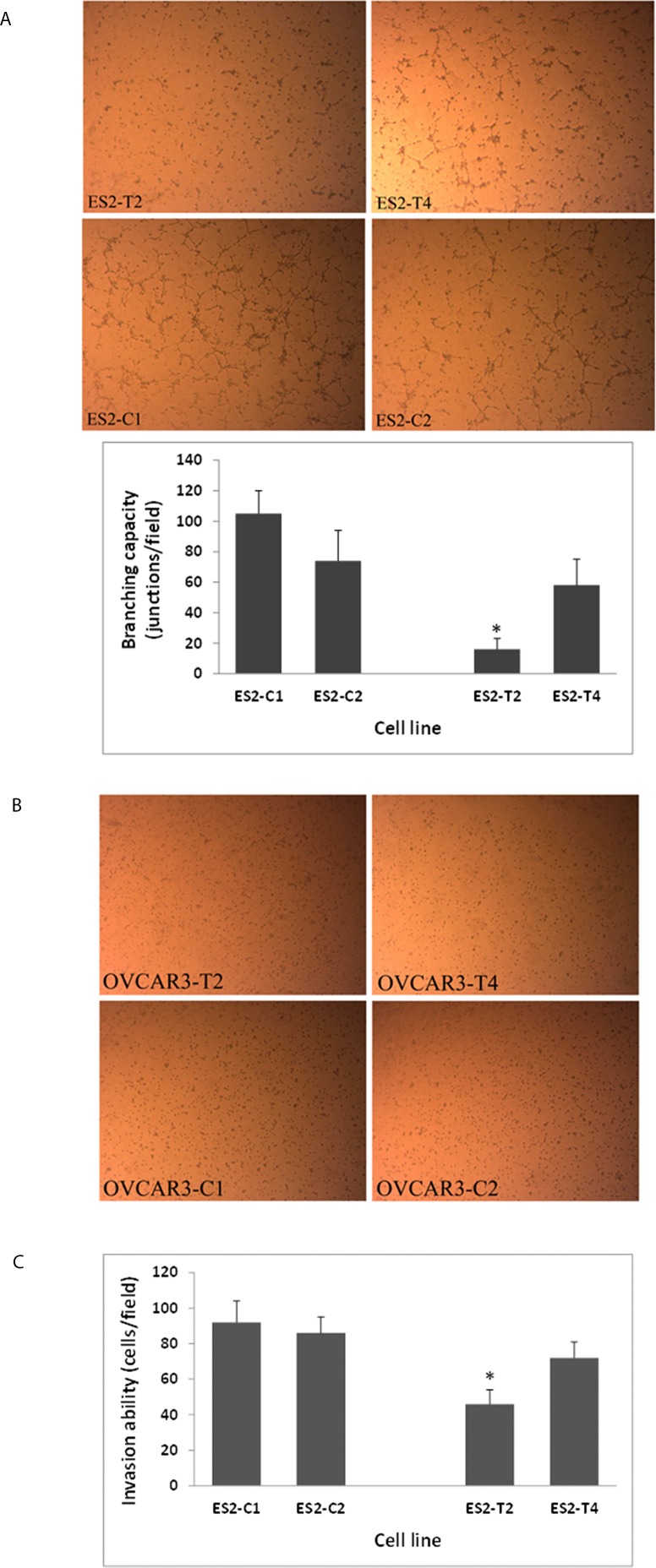
Reduced ezrin or p130Cas expression in ES2 cells resulted in decreased cell branching capacity on Matrigel and reduced invasion ability. (A) ES2 cells formed branches on Matrigel: ES2-C1 and ES2-C2, cells transfected with no plasmid and non-transfected cells, respectively. ES2-T4 cells, expressing reduced p130Cas, formed 35% less branches and the effect of reduced ezrin expression was more pronounced, forming 82% less branches (*, p<0.01 vs. ES2-C1 and p<0.05 vs. ES2-C2), as presented in the graph; (B) OVCAR3 cells remained dispersed on Matrigel regardless of ezrin or p130Cas expression: OVCAR3-C1 and OVCAR3-C2, cells transfected with no plasmid and non-transfected cells, respectively; OVCAR3-T2 and OVCAR3-T4 are cells expressing reduced of ezrin or p130Cas, respectively. In C, Cells that traversed the Matrigel-coated filters were stained and counted. ES2 cells with reduced ezrin expression (ES2-T2) showed 50% decrease in invasion ability compared the cells transfected with no plasmid (ES2-C1) and non-transfected cells (ES2-C2) (*, p<0.01). The effect in cells with reduced p130CAs expression (ES2-T4) was less pronounced, demonstrating 22% decrease in the invasion ability (Magnification X10).

ES2-C1 and ES2-C2 cells plated on Matrigel formed branches (Fig 4A). This feature was not seen in OVCAR3 cells, and the cells remained dispersed at the end of the incubation time in the control cells as well as in the transfected cells (Fig 4B). ES2-T4 cells, expressing reduced p130Cas, formed 35% less branches than the cells transfected with no plasmid (ES2-C1) and non-transfected cells (ES2-C2) when plated on Matrigel (Fig 4A). The effect of reduced ezrin expression in these cells was more pronounced. When ES2-T2 cells with reduced ezrin expression were plated on Matrigel, there was 82% less branching (p<0.01 vs. ES2-C1 and p<0.05 vs. ES2-C2), suggesting that the reduction in ezrin expression impaired the ability of the cells to migrate and remodel their microenvironment to form branches (Fig 4A).

### Ezrin and p130cas expression affects invasion through Matrigel

Decreased expression of Ezrin or p130Cas impaired the invasive capacity of ES2 cells through Matrigel. The ability of ES2-T2 cells with reduced ezrin expression to traverse Matrigel-covered filters was reduced by 50% compared to cells transfected with no plasmid (ES2-C1) and non-transfected cells (ES2–C2) (Fig 4C) (p<0.01). The effect of p130Cas reduced expression was less pronounced, and the invasive capacity of these cells (ES2-T4) was reduced only by 22% compared to control cells (ES2-C1 and ES2- C2; Fig 4C). OVCAR3 cells did not show any invasive capacity in this assay.

### Associations between ezrin and p130Cas expressions and clinicopathologic parameters

Analysis of the association between Ezrin and p130cas expression in effusions and clinicopathologic parameters showed overexpression of p-ERM protein in peritoneal compared to pleural effusions (p = 0.024), higher Ezrin protein levels in effusions from patients who had suboptimal debulking (p = 0.017) and overexpression of p130cas protein in post-chemotherapy effusions compared to effusions tapped pre-chemotherapy at diagnosis (p = 0.032). No significant associations were observed between RNA or protein levels and patient age, FIGO stage, CA 125 levels at diagnosis, response to chemotherapy at diagnosis or intrinsic chemoresistance (p>0.05), (Table 2).

**Table 2:** Significant associations between Ezrin and p130cas expression and clinicopathologic parameters.

Parameter	Ezrin mRNA	p130cas mRNA	p-Ezrin protein	Ezrin protein	p130cas protein
Specimen site (pleura vs. peritoneum)	p = 0.538	p = 0.245	**p = 0.024** ^*a*^	p = 0.859	p = 0.344
Previous chemotherapy (yes vs. no)	p = 0.351	p = 0.363	p = 0.387	p = 0.653	**p = 0.032** ^*b*^
Age (≤60 vs. >60 years)	p = 0.492	p = 0.843	p = 0.252	p = 0.599	p = 0.751
Residual disease (≤1 cm vs. >1 cm)	p = 0.263	p = 0.845	p = 0.735	**p = 0.017** ^*c*^	p = 0.331
Chemoresponse after primary treatment (complete vs. other)	p = 0.271	p = 0.412	p = 0.986	p = 0.660	p = 0.090
Primary chemoresistance (recurrence at ≤6 months)	p = 0. 391	p = 0.902	p = 0.577	p = 0.486	p = 0.529

^*a*^ Overexpression of p-ERM protein in peritoneal compared to pleural effusions

^*b*^ Overexpression of p130cas protein in post-chemotherapy effusions compared to effusions tapped pre-chemotherapy at diagnosis (p = 0.032).

^*c*^ higher Ezrin protein levels in effusions from patients who had suboptimal debulking

## Discussion

Ezrin and p130Cas are both cytosolic structural proteins that play an important role in signaling pathways affecting the cytoskeleton and regulating cell motility and proliferation, and are involved in cancer dissemination [1,2,7,9,28,29]. Although ezrin and p130Cas expression was studied to some extent in OC, to the best of our knowledge, no data is available regarding the expression in OC malignant effusions. In this study we observed higher expression of ezrin, with no significant change in p130Cas expression, in malignant effusions compared to solid tumors in both *in vitro* 3-dimensional cell culture models and in clinical HGSC samples. shRNA silencing in OC cell lines showed the involvement of ezrin more than p130Cas in processes that include ECM remodeling which is essential for tumor dissemination.

The majority of the *in vitro* studies use 2-dimensional cell cultures that do not mimic the 3-dimensional growth of the solid tumor in the body. Moreover, cells in malignant effusions survive and proliferate in very unique conditions, as anchorage-independent cells in clusters [22]. In the current study, the inert alginate scaffold enabled cells to form 3-dimensional structure of tumor cells with their surrounding microenvironment, modeling the growth of the ovarian tumor and solid metastases in the body. Rocking culture dish resulted in anchorage-independent cell proliferation forming suspended clusters, as in malignant effusions. Culture conditions may affect protein expression [30–33], and ezrin expression was higher in the cells in spheroids compared to the cells on the alginate scaffolds, with no change in p130Cas expression. These *in vitro* results were in line with our clinical series results, in which ezrin expression was significantly higher in malignant effusions compared to solid tumors, and no change was detected in p130Cas expression. This concurrence strengthens the validity of these 3-dimensional cell cultures as suitable models for solid tumor and malignant effusions in cancer research.

Ezrin or p-ezrin overexpression has been found in diverse human cancers and was associated with tumor progression and malignant phenotype [5–11]. Studies of the role of ezrin in OC have focused on solid tumors and found association with advanced disease [23,24]. Higher ezrin expression was found in OC metastases compared to primary tumor or normal ovary, but histotype was not specified [23]. Detection of ezrin in OC samples was correlated with reduced OS and in multivariate analysis, ezrin was found to be an independent prognostic factor [24], although in another study, negative or weak ezrin immunoreactivity in solid tumors was correlated with poor serous OC patient survival [34]. In the current study, Ezrin and p130Cas expression in effusions was unrelated to clinical outcome. The cohort of patients with effusions is unique, as it consists almost exclusively of patients diagnosed at FIGO stage IIIC or IV, which have relatively uniform outcome. The significantly higher expression of ezrin in effusions nevertheless suggests a possible role for this molecule in the transition from solid tumor to effusion. As the latter is a chemoresistant niche, this may have clinical implications. Phosphorylation of ezrin on Thr567 is considered as activation of the dormant linker protein [2,4], although more functions of ezrin that are unrelated to phosphorylation were reported [7]. Detection of Thr567 p-ezrin may be challenging since many antibodies also react with other phosphorylated ERM family members [7,9], as the antibody that was used in the current study. Radixin expression was not detected in the clinical samples, indicating that p-ezrin is the relevant protein in HGSC. Since no significant change was seen in phosphorylated ERM proteins in malignant effusions compared to the solid tumors, further research in needed to evaluate the specific role of each phosphorylated ERM protein in OC.

The two cell lines used in the in vitro assays are not optimal as HGSC models in view of the fact that they have other (ES2) or uncertain (OVCAR3) histology. Since they showed increased ezrin expression when cultured as spheroids in comparison to scaffolds, similarly to the clinical samples, and malignant effusions are characteristic of clear cell carcinoma as well, both cell lines were used in the in vitro assays for studying the contribution of ezrin to tumor progression.

Silencing ezrin in OC cells reduced their malignant phenotype. Reductions in invasion ability and cell branching capacity were pronounced, although no significant change could be seen in spheroid morphology or in the 24h proliferation rate. Similar results were reported by others in various cancer cell lines. Reduced expression of ezrin resulted in a decreased invasion capacity of breast cancer cells and a change in their morphology as spheroids on Matrigel [12], a significant reduction in proliferation, migration and invasion of lung adenocarcinoma cells [10], and a suppressed penetration of endometrial cancer cells through Matrigel membrane with no effect on proliferation rate [35]. It seems that although the effect on cell proliferation was not always detected, the involvement of ezrin in invasion was consistently shown. Our data and studies of other investigators suggest impaired ability of cells with reduced ezrin expression in processes that include remodeling of their surrounding extracellular matrix, which is a crucial step in tumor dissemination [36–38]. Ezrin overexpression promotes migration and invasion of cancer cells [2], and ezrin appears to allow metastatic cells to overcome a number of stresses experienced during the metastatic cascade, most notably, the stress experienced as cells interact with microenvironment of the secondary site [7,13]. Our results are in line with these studies. Ezrin functions as a linker between the plasma membrane and the cytoskeleton and is involved in cell motility, proliferation and the ability to survive anoikis [2,7,10,13], processes that are essential for cells in malignant effusions. The site-specific changes in ezrin expression together with the in vitro results point towards a role for increased ezrin expression in the early steps of OC dissemination into effusions, involving cytoskeletal changes, ECM remodeling, invasion and anchorage-independent proliferation and survival.

The role of p130Cas in cancer was studied mainly in other malignancies. In solid OC samples, higher p130Cas expression was correlated with advanced disease and worse prognosis [25]. In the current study, p130Cas expression was similar in the ovarian HGSC, solid metastases and malignant effusion. Notably, the phosphorylated form of p130Cas was barely detected in the clinical specimens regardless of tumor site, although clear bands were seen in the cell lines analyzed. Phosphorylation regulates p130Cas and results in protein translocation to the cell membrane and association with adhesion proteins [1,14]. A study by Patwardhan et al. showed that at least one effect of p130Cas on tumor malignancy is independent of phosphorylation and may result from a scaffolding function of the protein [14]. It is possible that the phosphorylated forms of p130Cas that were evaluated in the current study may not be involved in OC, yet more research is needed to find whether other phosphorylated tyrosines are more relevant.

Studies on the role of p130Cas in cancer showed that downregulation of the protein reduced the malignant phenotype of the cells. Silencing p130Cas in a breast cancer cell line resulted in reduced invasive capacity of the cells and the formation of smaller spheroids on Matrigel [12]. Silencing p130Cas expression in OC cell lines decreased tumor cell proliferation and angiogenesis and increased tumor cell death [25]. In the current study, silencing p130Cas in OC cell lines reduced the malignant phenotype of the cells, as evidenced by decreased invasive ability and reduced cell branching capacity upon Matrigel. However, the effect was less prominent in comparison to the marked effect of ezrin silencing. Our findings (clinical and in vitro) suggest that in contrast to other malignancies, p130Cas may not be a relevant therapeutic target in HGSC.

Ovarian and breast carcinomas share the ability to disseminate by forming malignant effusions. However, malignant effusions in breast carcinoma are associated with poorer prognosis compared to OC [39,40]. A previous study from our group found that in breast carcinoma effusions both ezrin and p130Cas were significantly overexpressed compared to primary carcinomas [20]. It seems that both types of malignant effusions, originating from different tumors, share higher ezrin, but not higher p130Cas expression. The significantly elevated ezrin expression in effusions of both breast and ovarian carcinomas points out towards ezrin as a possible target for therapeutic intervention against malignant effusions.

## Supporting Information

S1 Fig Clinical samples. Expression of ezrin, p-ezrin, p130cas and p-p130cas in clinical samples
(PDF)

S2 Fig Transfected cell lines. Expression of ezrin, p-ezrin, p130cas and p-p130cas in ES-2 and OVCAR3 cell lines.
(PDF)

## References

[1] Barrett A, Pellet-Many C, Zachary IC, Evans IM, Frankel P. p130Cas: a key signalling node in health and disease. Cell Signal. 2013;25:766–77. 10.1016/j.cellsig.2012.12.019 23277200

[2] Clucas J, Valderrama F. ERM proteins in cancer progression. J Cell Sci. 2014; 127:267–75. 10.1242/jcs.133108 24421310

[3] Adada M, Canals D, Hannun YA, Obeid LM. Sphingolipid regulation of ezrin, radixin, and moesin proteins family: implications for cell dynamics. Biochim Biophys Acta. 2014;1841:727–37. 10.1016/j.bbalip.2013.07.002 23850862 PMC3888837

[4] Gautreau A, Louvard D, Arpin M. Morphogenic effects of ezrin require a phosphorylation-induced transition from oligomers to monomers at the plasma membrane. J Cell Biol. 2000;150:193–203. 10893267 10.1083/jcb.150.1.193 PMC2185562

[5] Kong J, Li Y, Liu S, Jin H, Shang Y, Quan C, et al High expression of ezrin predicts poor prognosis in uterine cervical cancer. BMC Cancer. 2013;13:520 10.1186/1471-2407-13-520 24182314 PMC4228363

[6] Piao J, Liu S, Xu Y, Wang C, Lin Z, Qin Y, et al Ezrin protein overexpression predicts the poor prognosis of poor f pancreatic ductal adenocarcinomas. Exp Mol Pathol. 2015;98:1–6. 10.1016/j.yexmp.2014.11.003 25445504

[7] Ren L, Khanna C. Role of ezrin in osteosarcoma metastasis. Adv Exp Med Biol. 2014; 804:181–201. 10.1007/978-3-319-04843-7_10 24924175

[8] Jin T, Jin J, Li X, Zhang S, Choi YH, Piao Y, et al Prognostic implications of ezrin and phosphorylated ezrin expression in non-small cell lung cancer. BMC Cancer. 2014;14:191 10.1186/1471-2407-14-191 24629131 PMC3985600

[9] Bruce B, Khanna G, Ren L, Landberg G, Jirstorm K, Powell C, et al Expression of the cytoskeleton linker proteinezrin in human cancers. Clin Exp Metastasis. 2007;24:69–78. 17370041 10.1007/s10585-006-9050-x

[10] Li Q, Gao H, Xu H, Wang X, Pan Y, Hao F, et al Expression of ezrin correlates with malignant phenotype of lung cancer, and in vitro knockdown of ezrin reverses the aggressive biological behavior of lung cancer cells. Tumour Biol. 2012;33:1493–504. 10.1007/s13277-012-0400-9 22528947

[11] Gschwantler-Kaulich D, Natter C, Steurer S, Walter I, Thomas A, Salama M, et al Increase in ezrin expression from benign to malignant breast tumours. Cell Oncol (Dordr). 2013;6:485–91. 10.1007/s13402-013-0153-5 PMC13007475 24129929

[12] Konstantinovsky S, Davidson B, Reich R. Ezrin and BCAR1/p130Cas mediate breast cancer growth as 3 D spheroids. Clin Exp Metastasis. 2012;29:527–40. 10.1007/s10585-012-9468-2 22476538

[13] Heiska L, Melikova M, Zhao F, Saotome I, McClatchey AI, Carpén O. Ezrin is key regulator of Src-induced malignant phenotype in three-dimensional environment. Oncogene. 2011;30:4953–62. 10.1038/onc.2011.207 21666723

[14] Patwardhan P, Shen Y, Goldberg GS, Miller WT. Individual Cas phosphoryaltion sites are dispensable for processive phosphorylation by Src and anchorage-independent cell growth. J Biol Chem. 2006;281:20689–97. 16707485 10.1074/jbc.M602311200 PMC2441569

[15] Honda H, Oda H, Nakamoto T, Honda Z, Sakai R, Suzuki T, et al Cardiovascular anomaly, impaired actin bundling and resistance to Src-induced transformation in mice lacking p130Cas. Nat Genet. 1998;19:361–5. 9697697 10.1038/1246

[16] Nikonova AS, Gaponova AV, Kudinov AE, Golemis EA. CAS proteins in health and disease: an update. IUBMB Life. 2014;66:387–95. 10.1002/iub.1282 24962474 PMC4111207

[17] Hempel N, Bartling TR, Mian B, Melendez JA. Acquisition of the metastatic phenotype is accompanied by H2O2-dependent activation of the p130Cas signaling complex. Mol Cancer Res. 2013;11:303–12. 10.1158/1541-7786.MCR-12-0478 23345605 PMC3606285

[18] Tornillo G, Bisaro B, Camacho-Leal Mdel P, Galie M, Provero P, Di Stefano P, et al p130Cas promotes invasiveness of three-dimensional ErbB2-transformed mammary acinar structures by enhanced activation of mTOR/p70S6K and Rac1. Eur J Cell Biol. 2011;90:237–48. 10.1016/j.ejcb.2010.09.002 20961652

[19] Miao Y, Li AL, Wang L, Fan CF, Zhang XP, Xu HT, et al Expression of p130cas, E-cadherin and β-catenin and their correlation with clinicopathological parameters in non-small cell lung cancer: p130cas over-expression predicts poor prognosis. Folia Histochem Cytobiol. 2012;50:392–7. 10.5603/11957 23042269

[20] Konstantinovsky S, Smith Y, Zilber S, Stavens HT, Becker AM, Nesland JM, et al Breast carcinoma cells in primary tumors and effusions have different gene array profiles. J Oncol. 2010;2010:969084 10.1155/2010/969084 19680458 PMC2725284

[21] Siegel R, Ma J, Zou Z, Jemal A. Cancer statistics, 2014. CA Cancer J Clin. 2014;64:9–29. 10.3322/caac.21208 24399786

[22] Davidson B. Ovarian and Primary Peritoneal Carcinoma In Serous Effusions- Etiology, Diagnosis, Prognosis and Therapy. Edited by Davidson B, Firat P, Michael CM. London: Springer; 2011;167–204.

[23] Chen Z, Fadiel A, Feng Y, Ohtani K, Rutherford T, Naftolin F. Ovarian epithelial carcinoma tyrosine phosphorylation, cell proliferation, and ezrin translocation are stimulated by interleukin 1alpha and epidermal growth factor. Cancer. 2001;92:3068–75. 11753986 10.1002/1097-0142(20011215)92:12<3068::aid-cncr10149>3.0.co;2-5

[24] Köbel M, Gradhand E, Zeng K, Schmitt W, Kriese K, Lantzsch T, et al Ezrin promotes ovarian carcinoma cell invasion and its retained expression predicts poor prognosis in ovarian carcinoma. Int J Gynecol Pathol. 2006;25:121–30. 16633060 10.1097/01.pgp.0000185410.39050.ac

[25] Nick AM, Stone RL, Armaiz-Pena G, Ozpolat B, Tekedereli I, Graybill WS, et al Silencing of p130cas in ovarian carcinoma: a novel mechanism for tumor cell death. J Natl Cancer Inst. 2011;103:1596–612. 10.1093/jnci/djr372 21957230 PMC3206039

[26] Thigpen JT, Blessing JA, Ball H, Hummel SJ, Barrett RJ. Phase II trial of paclitaxel in patients with progressive ovarian carcinoma after platinum-based chemotherapy: a Gynecologic Oncology Group study. J Clin Oncol. 1994;12:1748–53. 7916038 10.1200/JCO.1994.12.9.1748

[27] Therasse P, Arbuck SG, Eisenhauer EA, Wanders J, Kaplan RS, Rubinstein L, et al New guidelines to evaluate the response to treatment in solid tumors. European Organization for Research and Treatment of Cancer, National Cancer Institute of the United States, National Cancer Institute of Canada. J Natl Cancer Inst. 2000;92:205–16. 10655437 10.1093/jnci/92.3.205

[28] Coene ED, Gadelha C, White N, Malhas A, Thomas B, Shaw M, et al A novel role for BRCA1 in regulating breast cancer cell spreading and motility. J Cell Biol. 2011;192:497–512. 10.1083/jcb.201004136 21282464 PMC3101087

[29] Cabodi S, del Pilar Camacho-Leal M, Di Stefano P, Defilippi P. Integrin signalling adaptors: not only figurants in the cancer story. Nat Rev Cancer. 2010;10:858–70. 10.1038/nrc2967 21102636

[30] Tatullo M, Marrelli M, Falisi G, Rastelli C, Palmieri F, Gargari M, et al Mechanical influence of tissue culture plates and extracellular matrix on mesenchymal stem cell behavior: A topical review. Int J Immunopathol Pharmacol. 2016;29:3–8. 10.1177/0394632015617951 26612837 PMC5806742

[31] Guiro K, Patel SA, Greco SJ, Rameshwar P, Arinzeh TL. Investigating breast cancer cell behavior using tissue engineering scaffolds. PLoS One. 2015;10:e0118724 10.1371/journal.pone.0118724 25837691 PMC4383476

[32] Sandercock AM, Rust S, Guillard S, Shachsenmeier KF, Holoweckyj N, Hay C, et al Identification of anti-tumour biologics using primary tumour models, 3-D phenotypic screening and image-based multi-parametric profiling. Mol Cancer. 2015;14:147 10.1186/s12943-015-0415-0 26227951 PMC4521473

[33] Bruce A, Evans R, Mezan R, Shi L, Moses BS, Martin KH, et al Three- Dimensional Microfluidic Tri-Culture Model of the Bone Marrow Microenvironment for Study of Acute Lymphoblastic Leukemia. PLoS One. 2015;10:e0140506 10.1371/journal.pone.0140506 26488876 PMC4619215

[34] Moilanen J, Lassus H, Leminen A, Vaheri A, Bützow R, Carpén O. Ezrin immunoreactivity in relation to survival in serous ovarian carcinoma patients. Gynecol Oncol. 2003;90:273–81. 12893187 10.1016/s0090-8258(03)00262-2

[35] Ohtani K, Sakamoto H, Rutherford T, Chen Z, Satoh K, Naftolin F, et al Ezrin, a membrane-cytoskeletal linking protein, is involved in the process of invasion of endometrial cancer cells. Cancer Lett. 1999;147:31–8. 10660086 10.1016/s0304-3835(99)00272-4

[36] Jacob A, Prekeris R. The regulation of MMP targeting to invadopodia during cancer metastasis. Front Cell Dev Biol. 2015;3:4 10.3389/fcell.2015.00004 25699257 PMC4313772

[37] Catalano V, Turdo A, Di Franco S, Dieli F, Todaro M, Stassi G. Tumor and its microenvironment: a synergistic interplay. Semin Cancer Biol. 2013;23:522–32. 10.1016/j.semcancer.2013.08.007 24012661

[38] Cho A, Howell VM, Colvin EK. The Extracellular Matrix in Epithelial Ovarian Cancer—A Piece of a Puzzle. Front Oncol. 2015;5:245 10.3389/fonc.2015.00245 26579497 PMC4629462

[39] Dieterich M, Goodman SN, Rojas-Corona RR, Emralino AB, Jimenez-Joseph D, Sherman ME. Multivariate analysis of prognostic features in malignant pleural effusions from breast cancer patients. Acta Cytol. 1994;38:945–52. 7992584

[40] Banerjee AK, Willetts I, Robertson JF, Blamey RW. Pleural effusion in breast cancer: a review of the Nottingham experience. Eur J Surg Oncol. 1994;20:33–6. 8131866

