# Supplementary material for: Ezrin Is Associated with Disease Progression in Ovarian Carcinoma
Source: PLoS One. 2016 Sep 13;11(9):e0162502. doi: 10.1371/journal.pone.0162502 (PMC5021292; doi:10.1371/journal.pone.0162502)

169

merge veredho 2015-03-19 12hr 02min+veredho 2015-03-19 11hr 58min_Exposure_189.8sec

Clinical samples

phospho ezrin 19.3.15

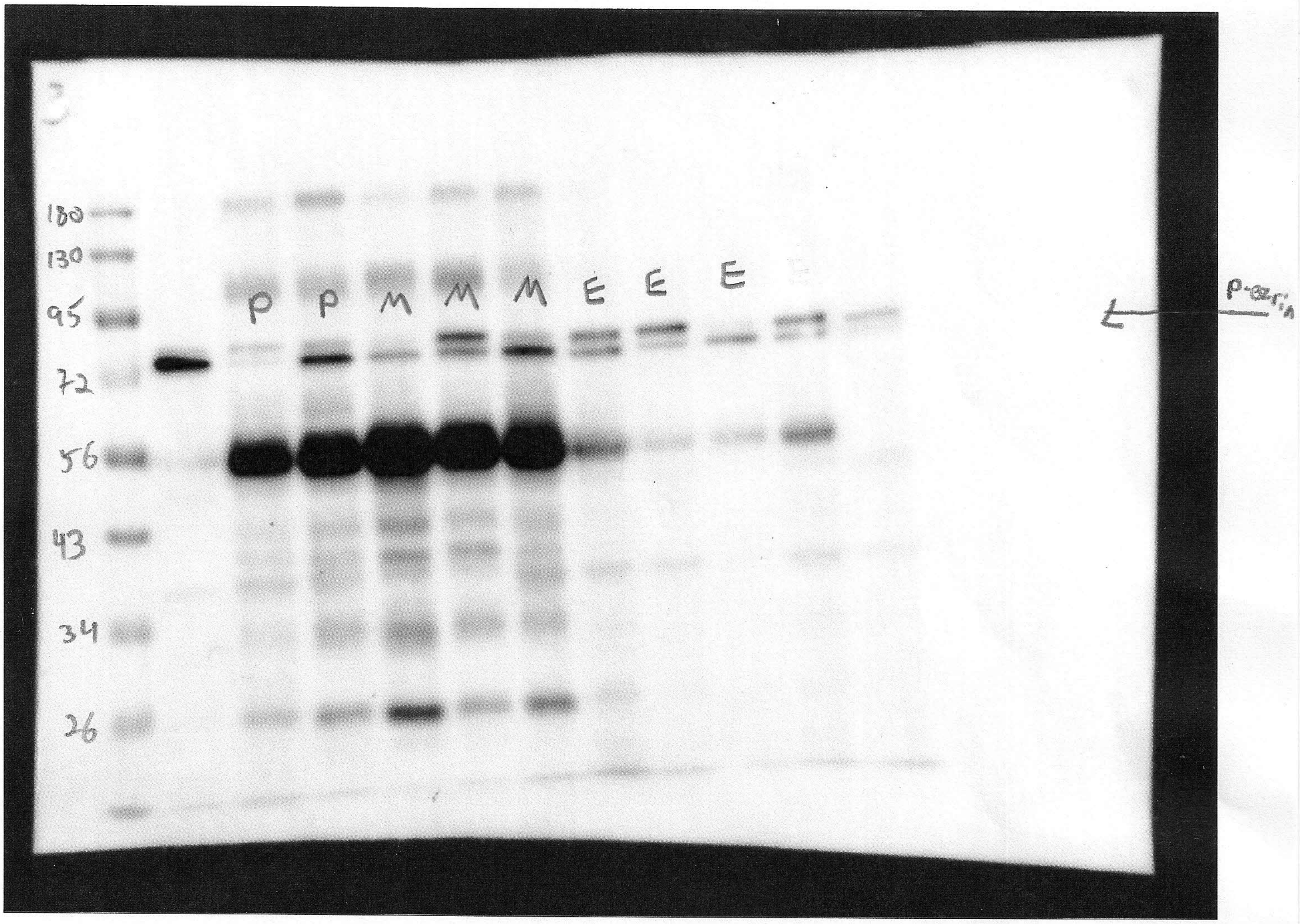

196

merge veredho 2015-03-23 11hr 42min+veredho 2015-03-23 11hr 41min_Exposure_5.0sec

clinical samples

GAPDH 22.3.15 (phospho ezrin 19.3.15)

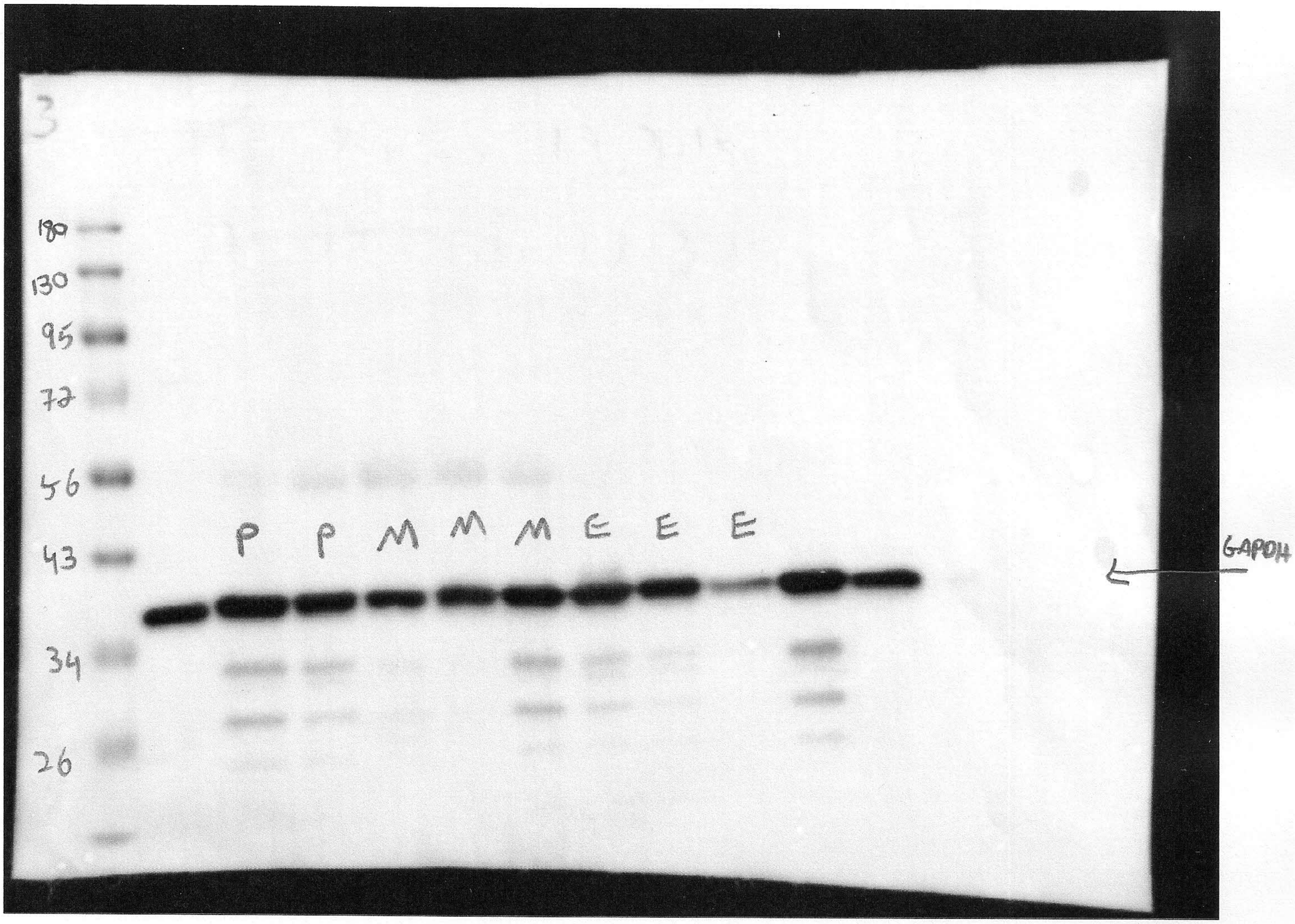

143

merge veredho 2015-02-16 11hr 19min_Exposure_600.0sec+veredho 2015-02-16 11hr 30min

Clinical samples

phospho ezrin 16.2.15

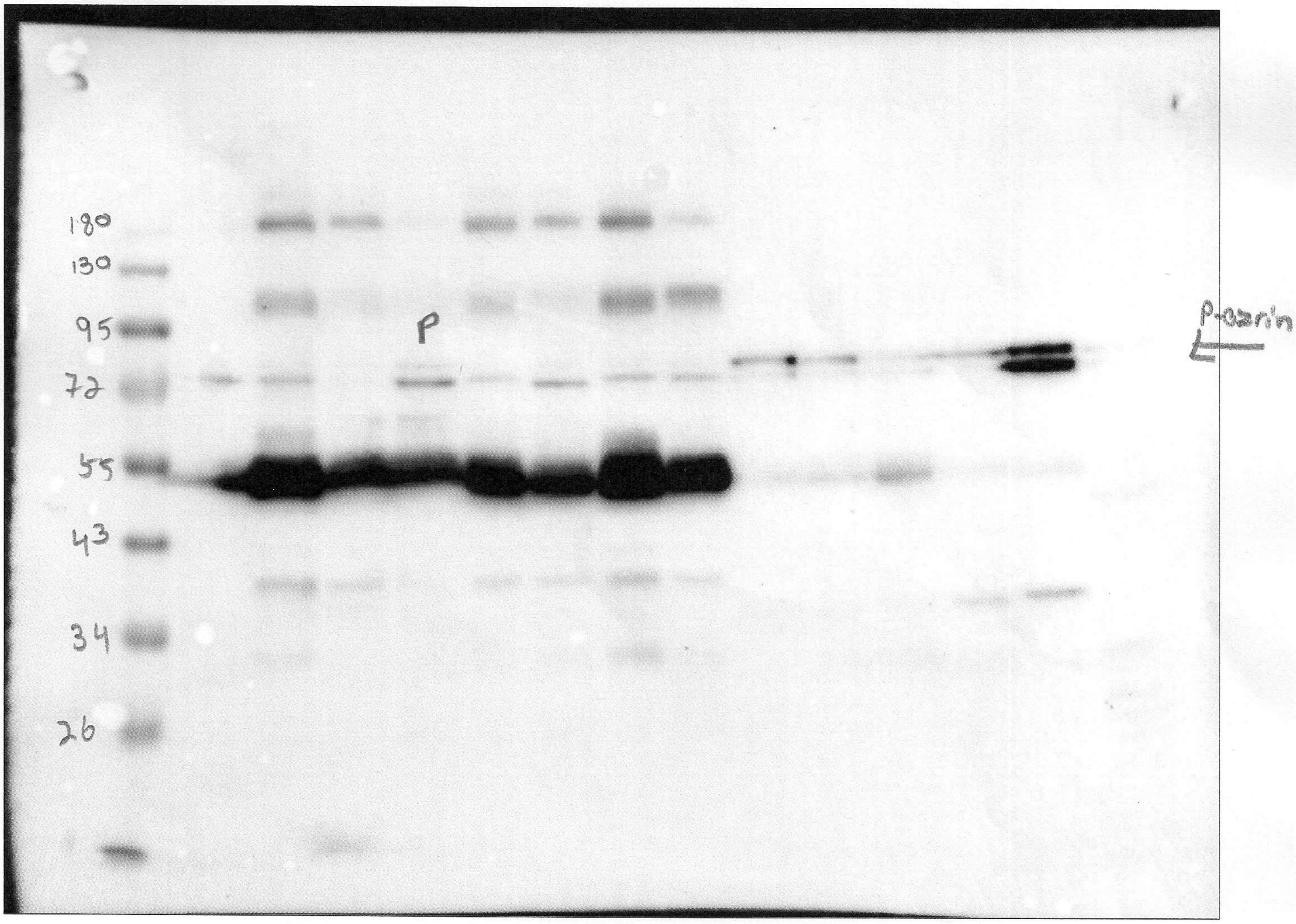

P:/labdata/reuvenr_lab_data/vered/vered 011014/western results/western ovaries/phospho ezrin/phospho ezrin 160215
Printed: 30/06/2016 14:50:15

173

merge veredho 2015-03-15 12hr 29min+veredho 2015-03-15 12hr 27min_Exposure_7.2sec

Clinical samplos

GAPDH 15.3.15 (phospho ezrin 16.2.15)

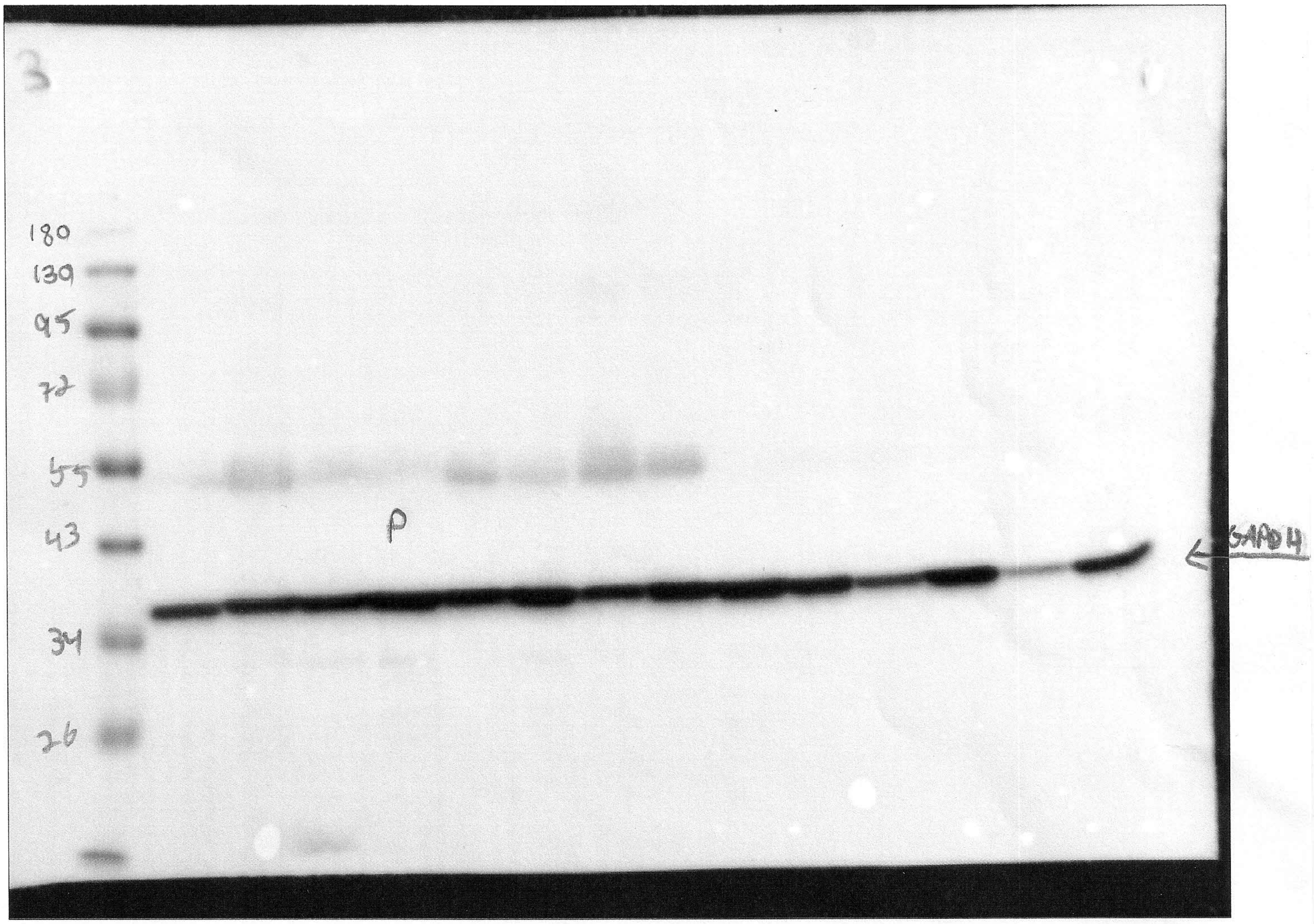

P:/labdata/reuvenr_lab_data/vered/vered 011014/west…s/western ovaries/GAPDH/GAPDH 150315/GAPDH 150315 3
Printed: 30/06/2016 14:55:04

68

merge veredho 2014-12-15 10hr 48min_Exposure_4.0sec+veredho 2014-12-15 10hr 50min

Clinical samplos

Ezrin 15.12.14

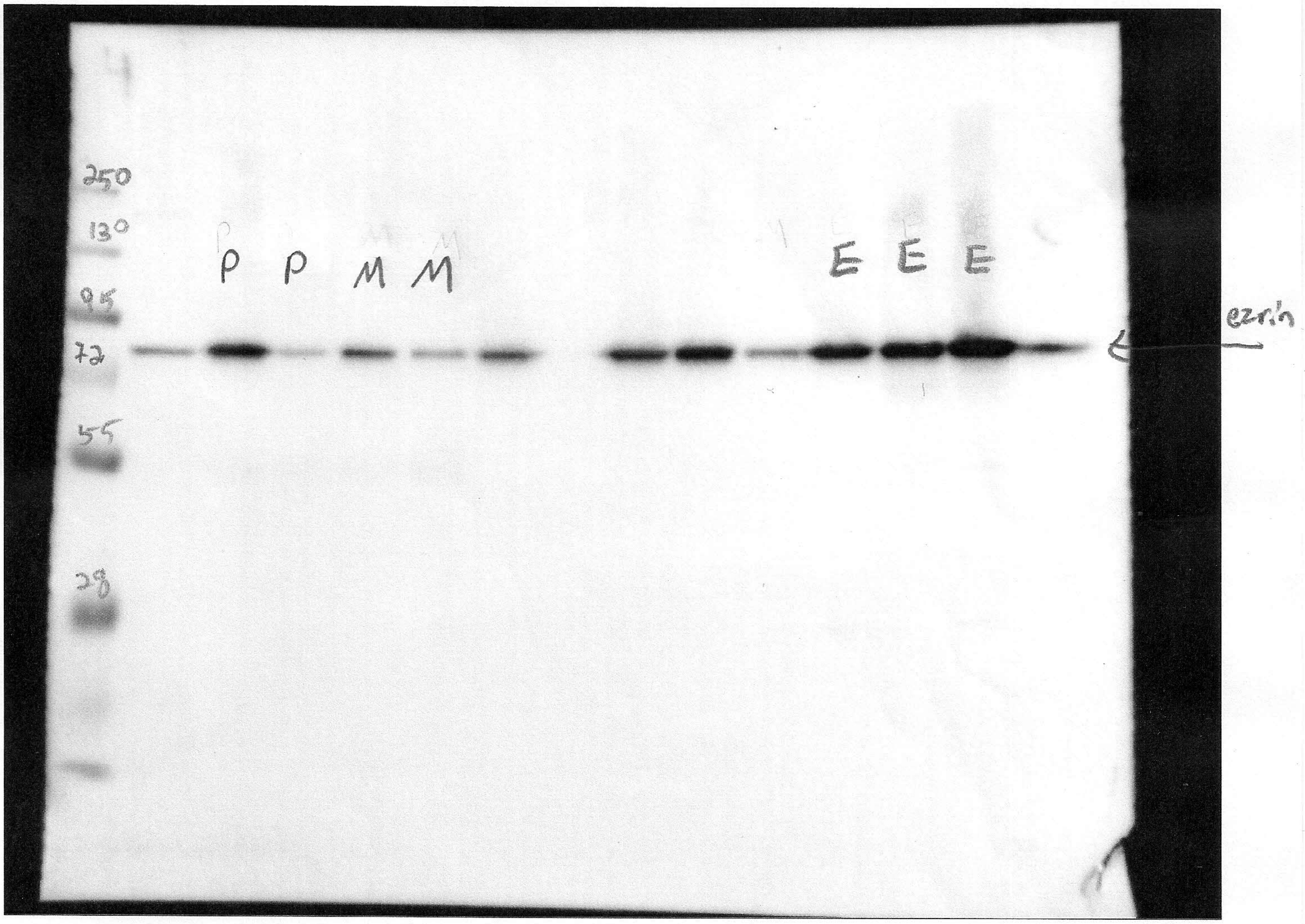

Location: P:/labdata/reuvenr_lab_data/vered/vered 011014/western results/western ovaries/ezrin/ezrin 151214
Printed: 30/06/2016 14:30:12

117

merge veredho 2015-01-28 11hr 53min+veredho 2015-01-28 11hr 51min_Exposure_60.0sec

Clinical samplos

GAPDH 28.1.15 (ezrin 15.12.14)

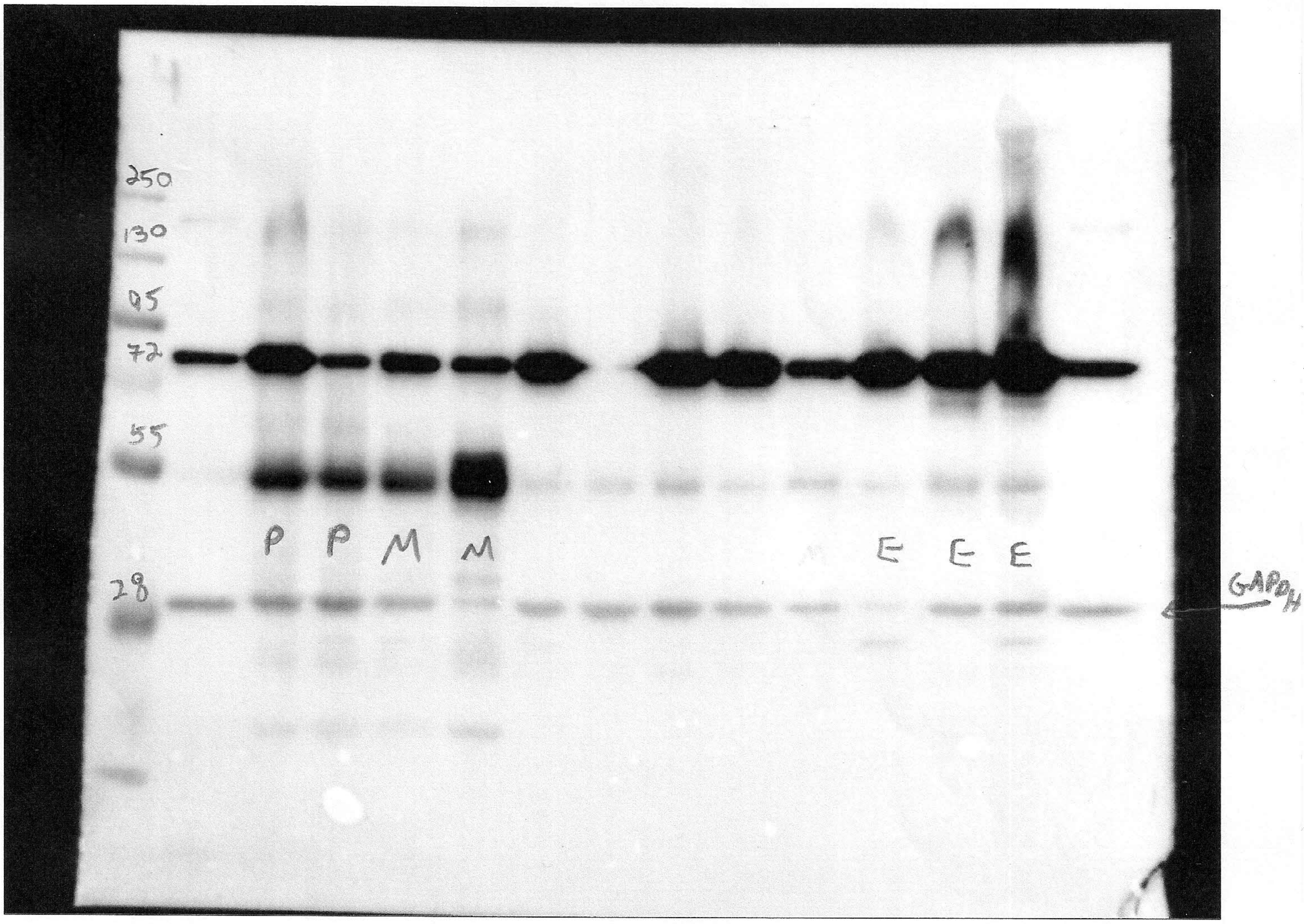

58

merge veredho 2014-12-02 11hr 08min+veredho 2014-12-02 11hr 06min_Exposure_2.9sec

Clinical Samples

Ezrin 2.12.14

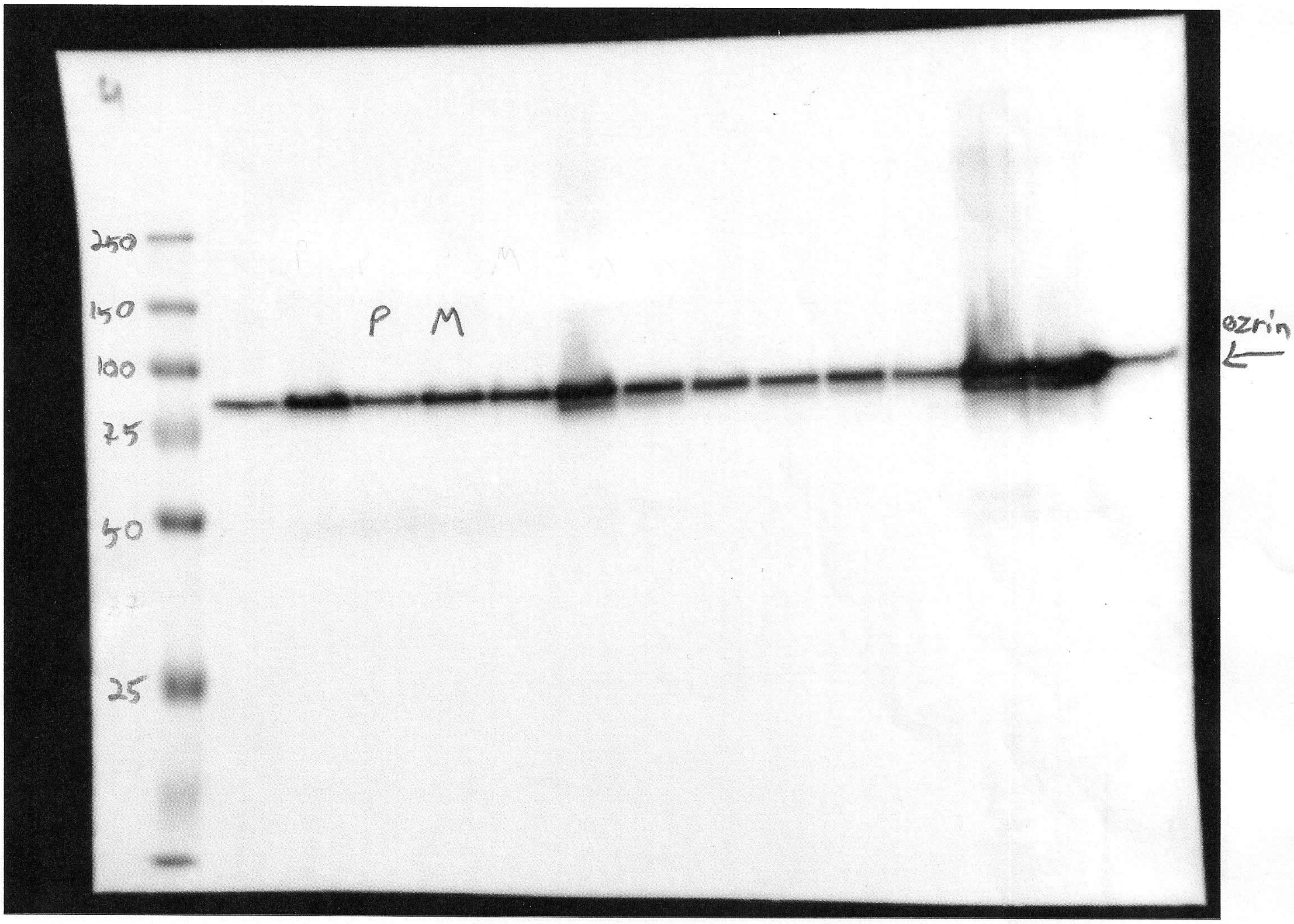

Location: P:/labdata/reuvenr_lab_data/vered/vered 011014/western results/western ovaries/ezrin/ezrin 011214
Printed: 30/06/2016 14:29:05

1/14

merge veredho 2015-01-27 12hr 10min+veredho 2015-01-27 12hr 07min_Exposure_60.0sec

clinical samples

GAPDH 27.1.15 (ezrin 2.12.14)

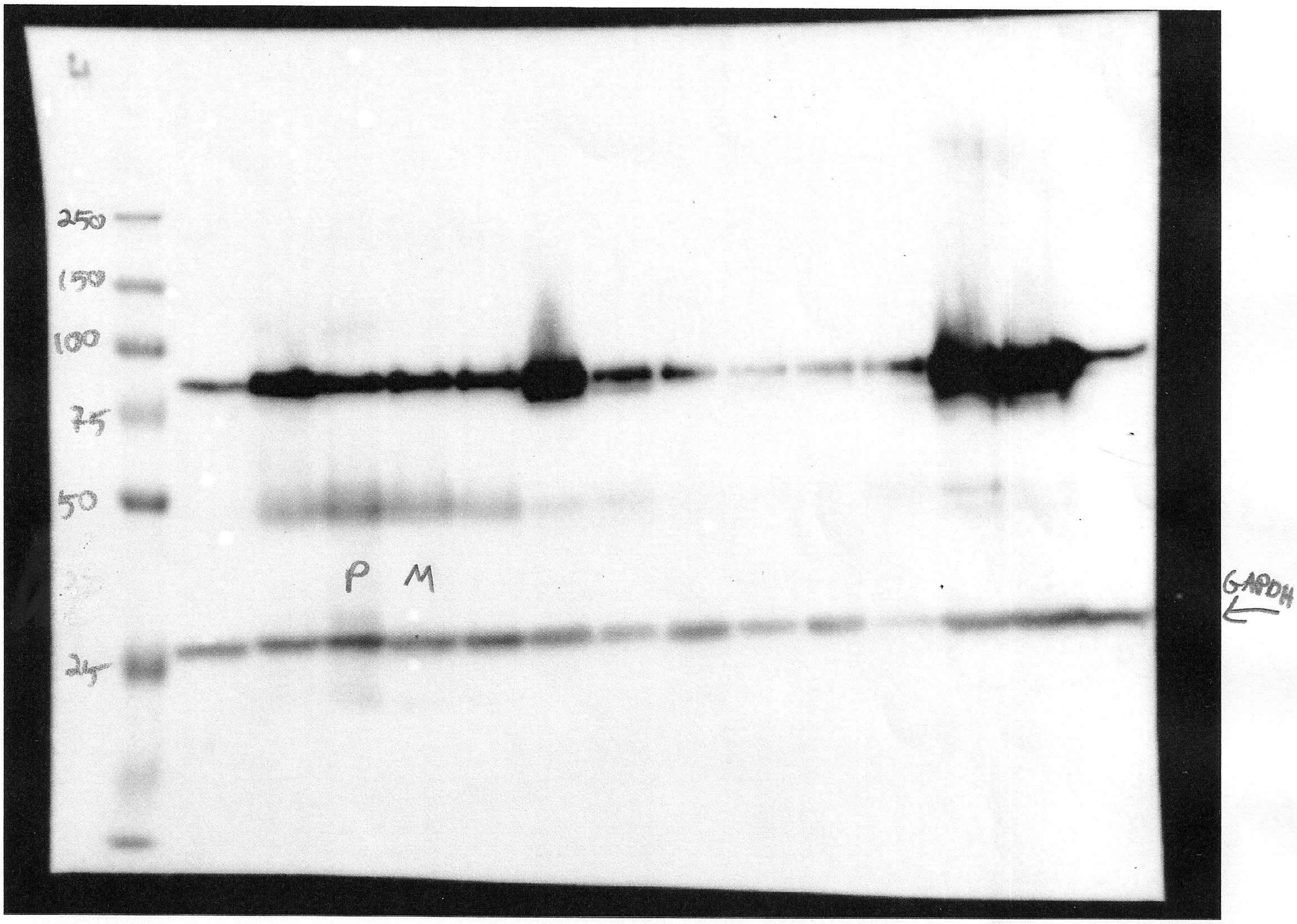

P:/labdata/reuvenr_lab_data/vered/vered 011014/west…s/western ovaries/GAPDH/GAPDH 270115/GAPDH 270115 4
Printed: 30/06/2016 14:48:04

merge veredho 2014-11-25 11hr 15min+veredho 2014-11-25 11hr 11min_Exposure_74.9sec

Clinical samples

p130Cas 25.11.14

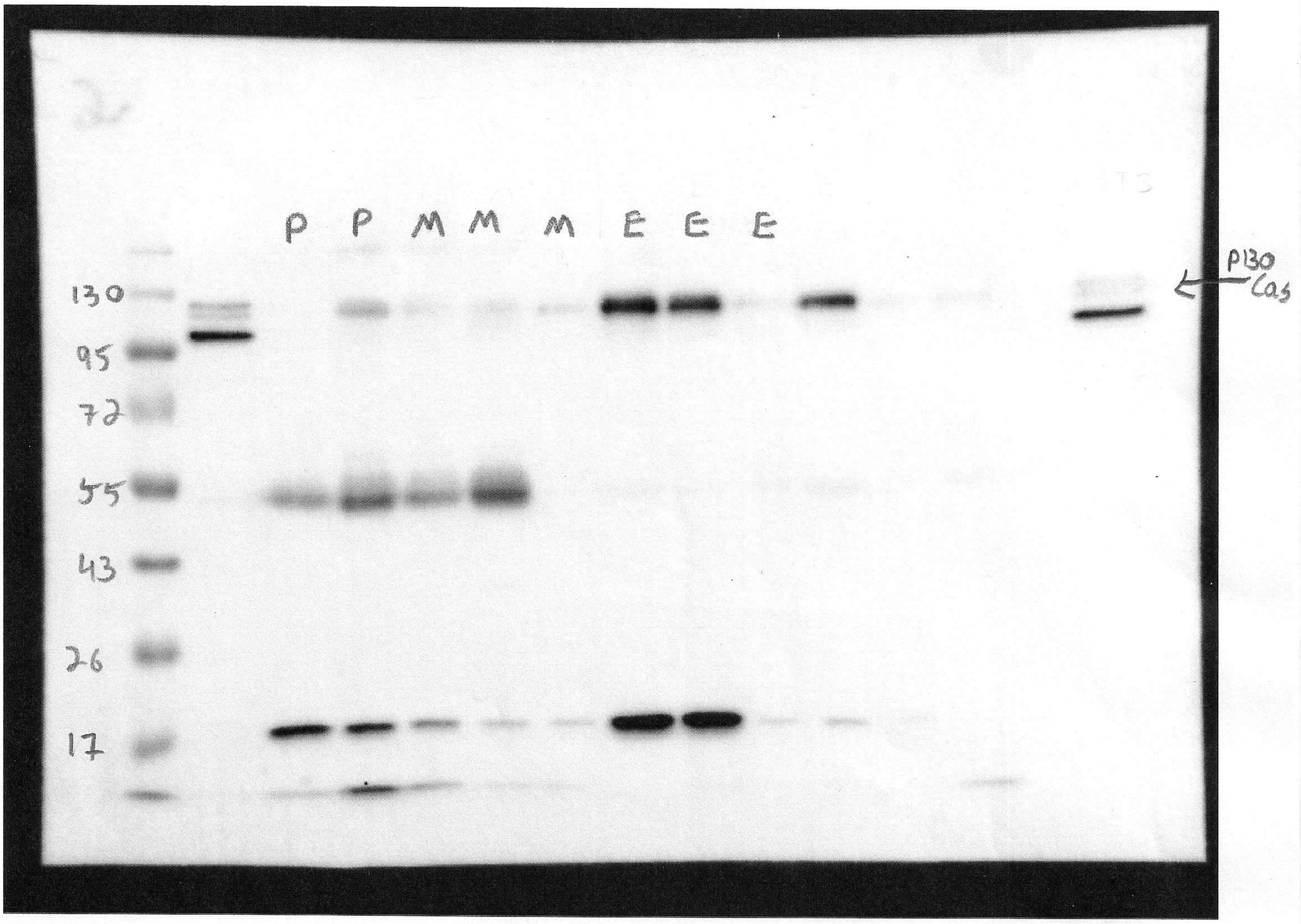

91

merge veredho 2014-12-29 11hr 25min_Exposure_17.1sec+veredho 2014-12-29 11hr 27min

clinical samples

GAPDH 29.12.14 (p130cas 25.11.14)

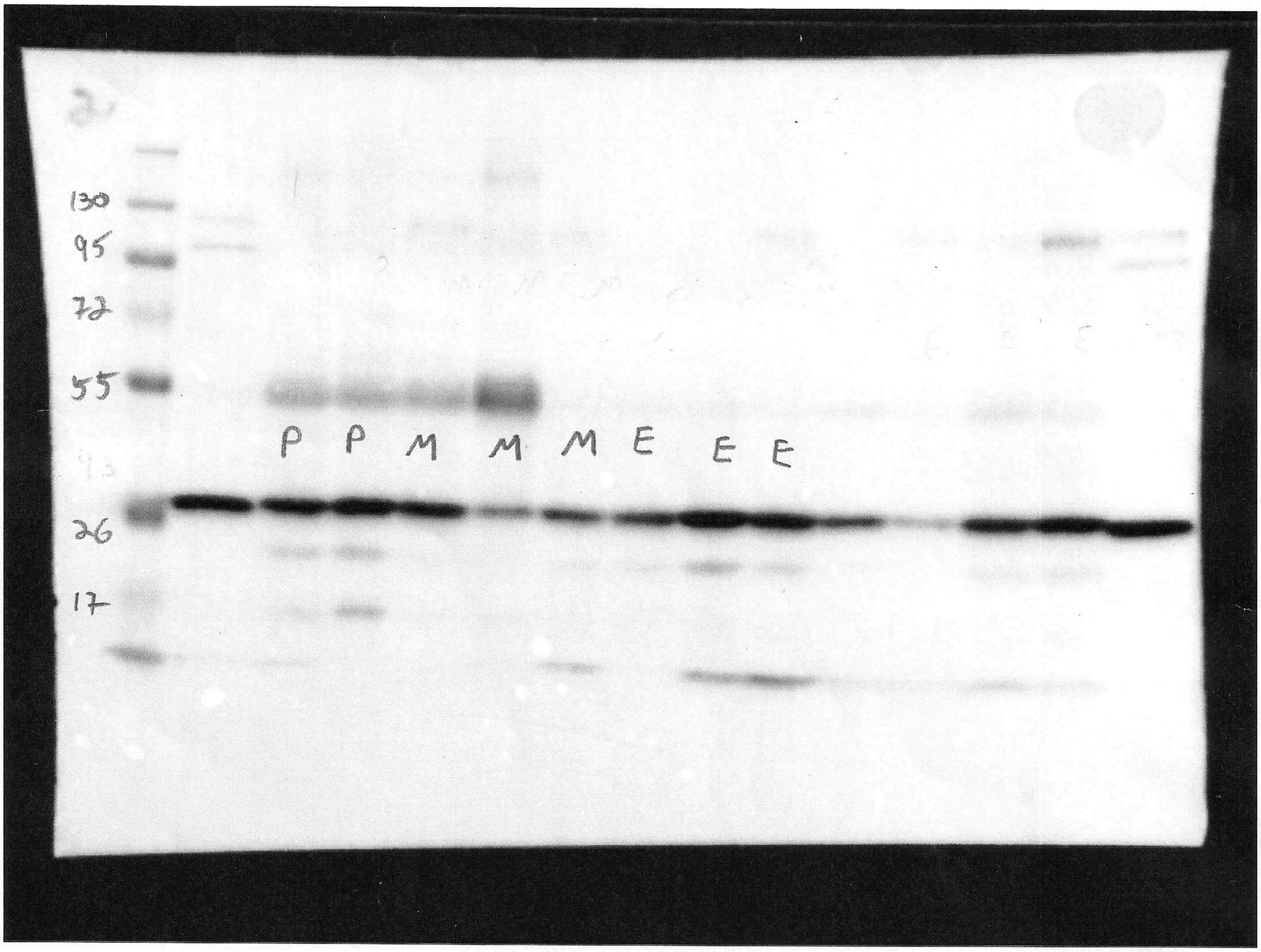

P:/labdata/reuvenr_lab_data/vered/vered 011014/west...s/western ovaries/GAPDH/GAPDH 291214/GAPDH 291214 1
Printed: 30/06/2016 14:24:40

merge veredho 2015-02-24 10hr 50min_Exposure_30.0sec+veredho 2015-02-24 10hr 55min

clinical samplos

p130Cas 24.2.15

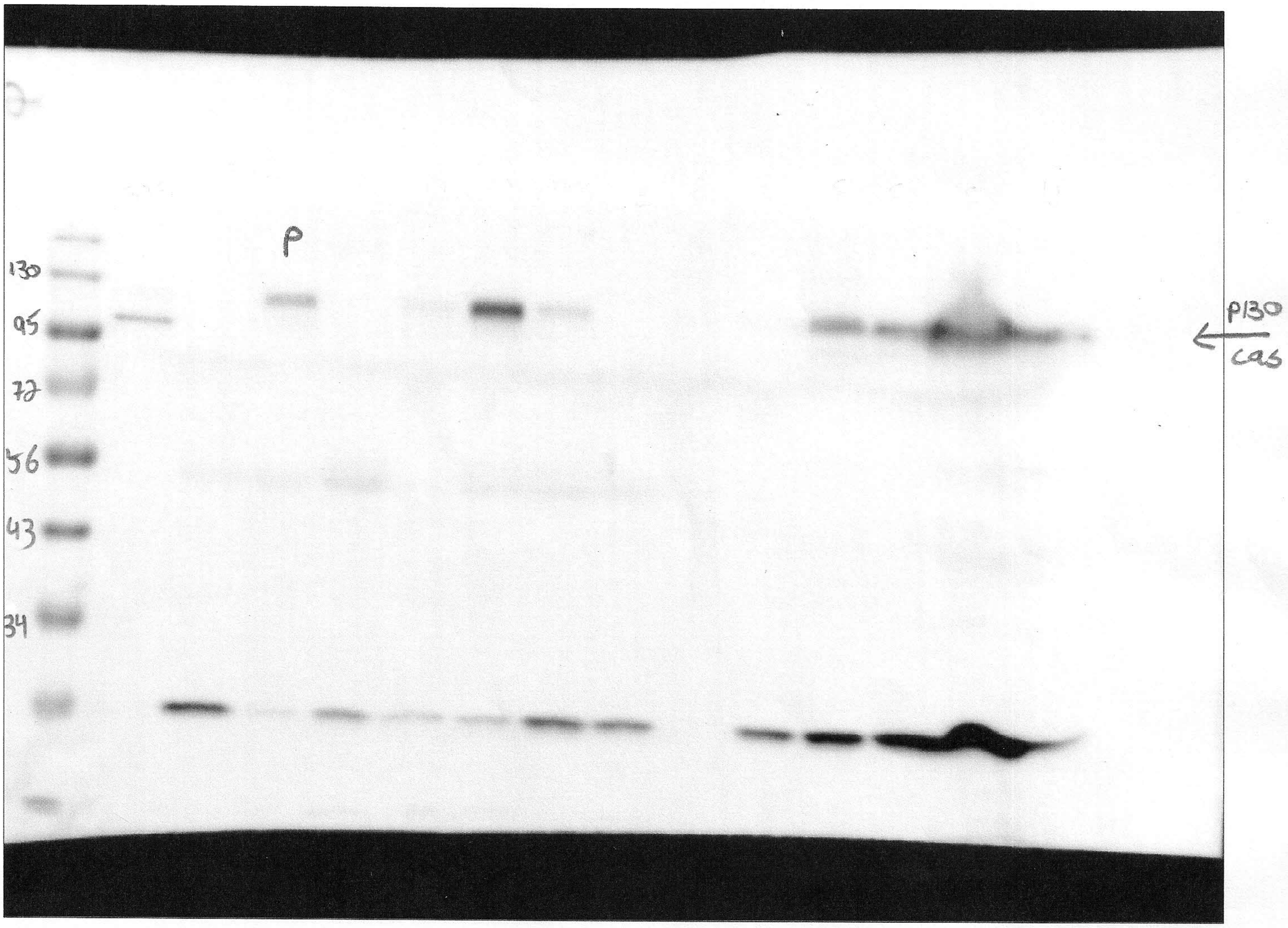

187

merge veredho 2015-03-18 10hr 56min+veredho 2015-03-18 10hr 53min_Exposure_8.0sec

Clinical samplos

GAPDH 18.3.15 (p130Cas 24.2.15)

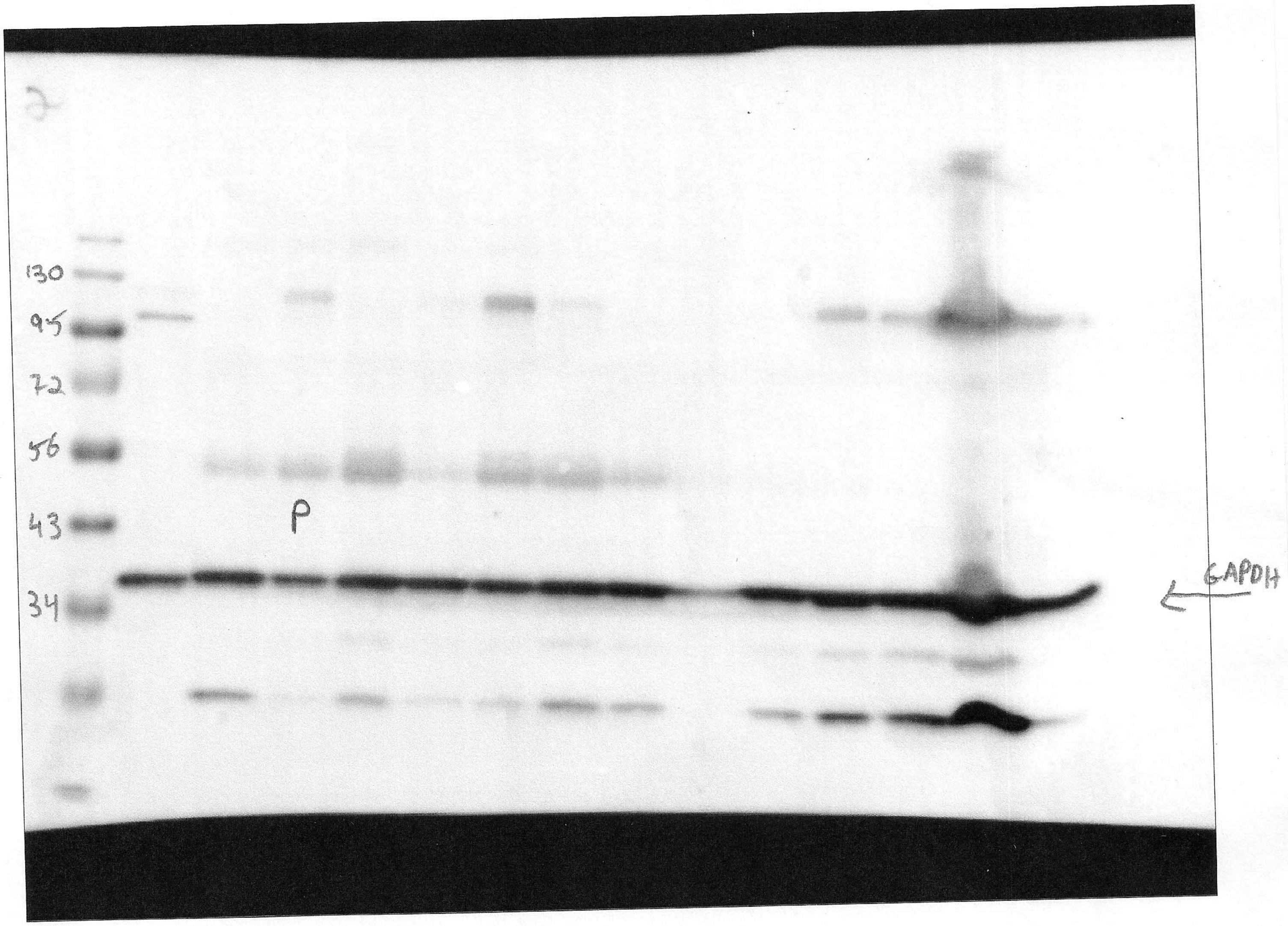

Supplement: S1 Fig — Expression of ezrin, p-ezrin, p130cas and p-p130cas in clinical samples (PDF) [file pone.0162502.s001.pdf]
