# Supplementary material for: Ezrin Is Associated with Disease Progression in Ovarian Carcinoma
Source: PLoS One. 2016 Sep 13;11(9):e0162502. doi: 10.1371/journal.pone.0162502 (PMC5021292; doi:10.1371/journal.pone.0162502)

merge veredho 2015-08-04 11hr 11min_Exposure_126.9sec+veredho 2015-08-04 11hr 17min

Transfected cell lines

ES2

phospho ERM 4.8.15

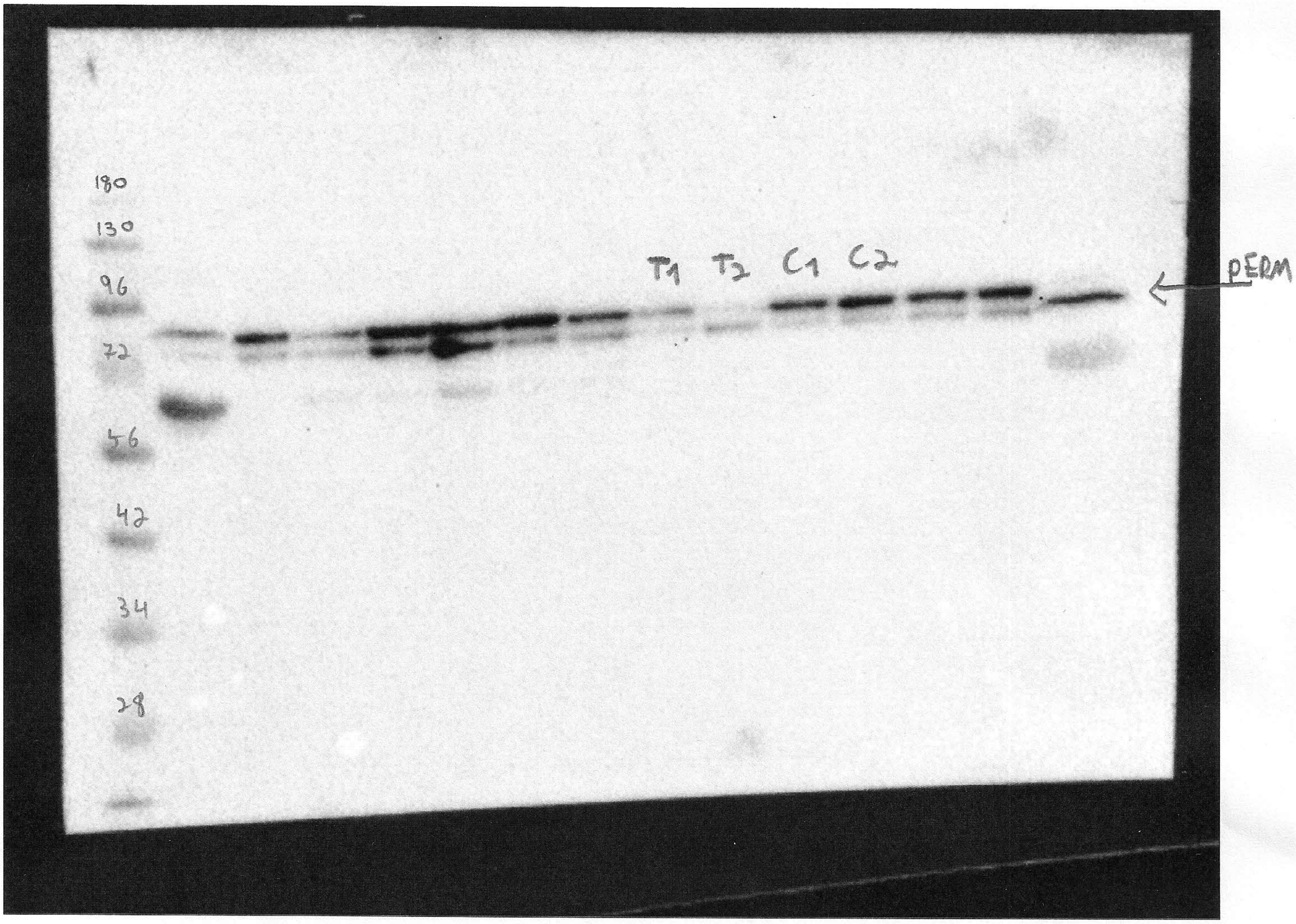

merge veredho 2015-08-17 14hr 17min_Exposure_6.0sec+veredho 2015-08-17 14hr 18min

Transfected cell lines
ES2
GAPDH (17.8.15 (phospho ERM 4.8.15)

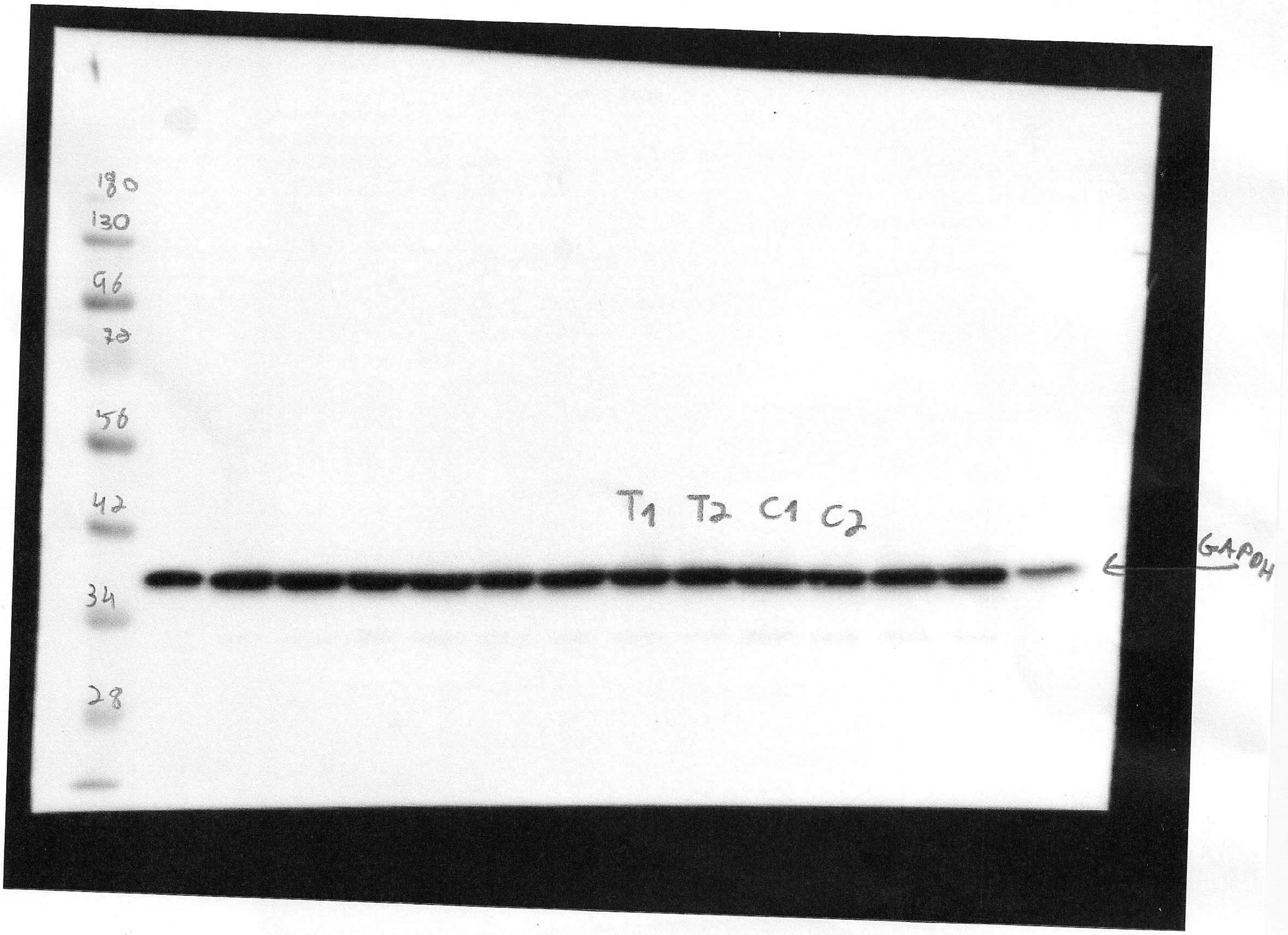

merge veredho 2015-08-04 11hr 28min_Exposure_8.4sec+veredho 2015-08-04 11hr 30min

Transfected cell lines
ES2
Ezrin 4.8.15

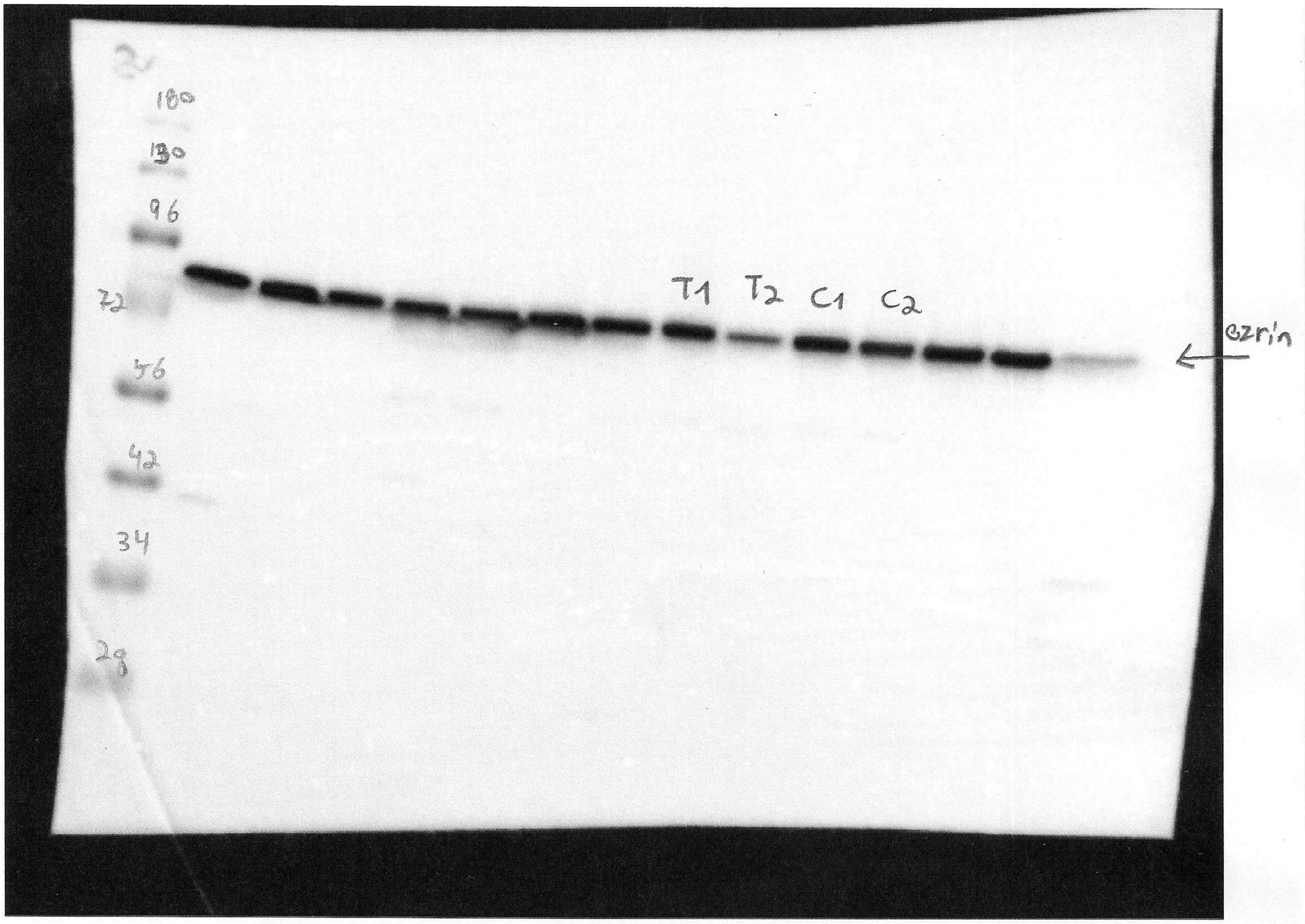

Location: P:/labdata/reuvenr_lab_data/vered/vered 011014/western results/cell lines transfections/ezrin/ezrin 040815
Printed: 30/06/2016 12:24:37

merge veredho 2015-08-17 14hr 19min_Exposure_4.0sec+veredho 2015-08-17 14hr 21min

Transfected cell lines
ES2
GAPDH 17.8.15 (ezrin 4.8.15)

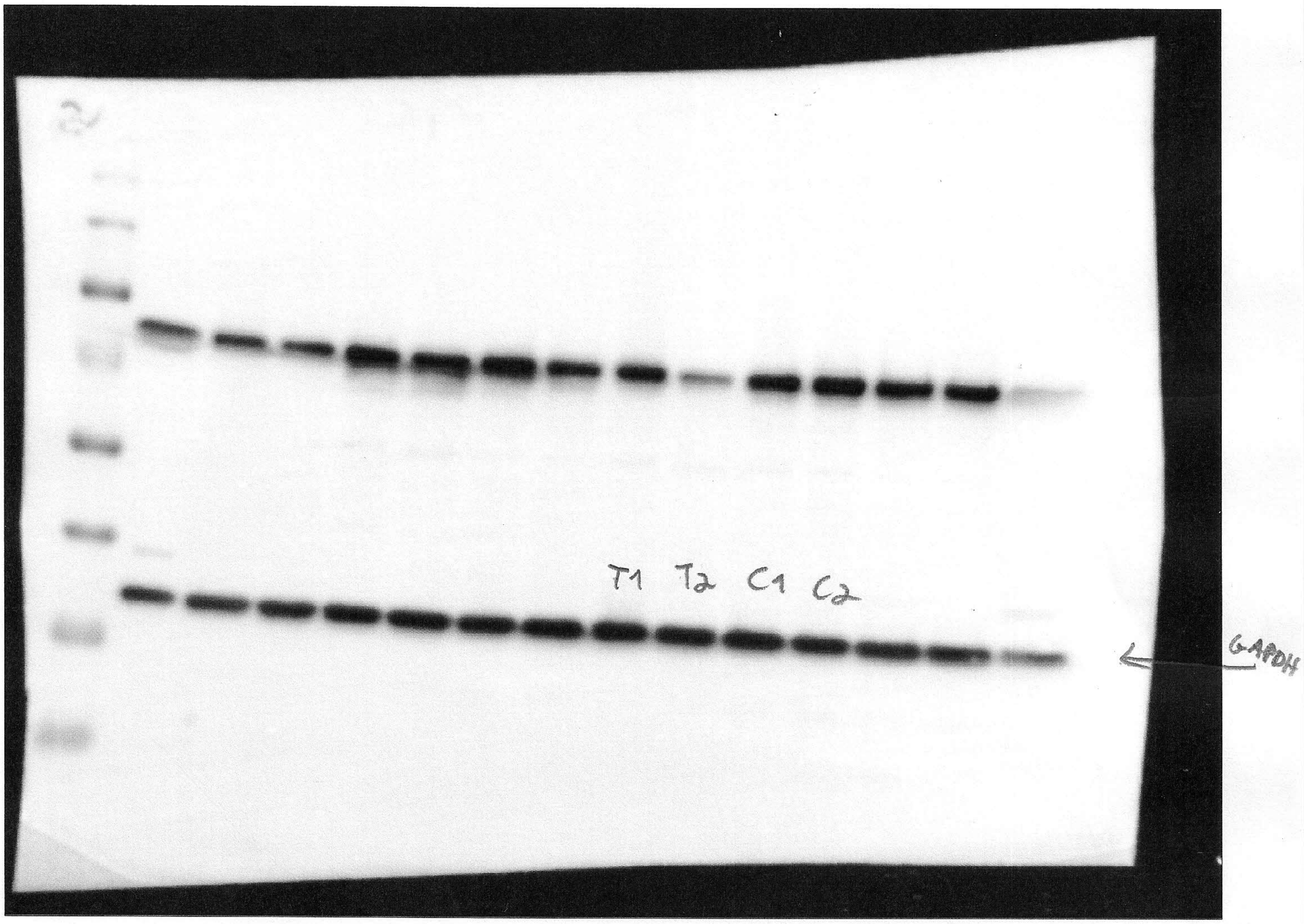

Transfected cell lines

ES2

phospho p130Cas 4.8.15

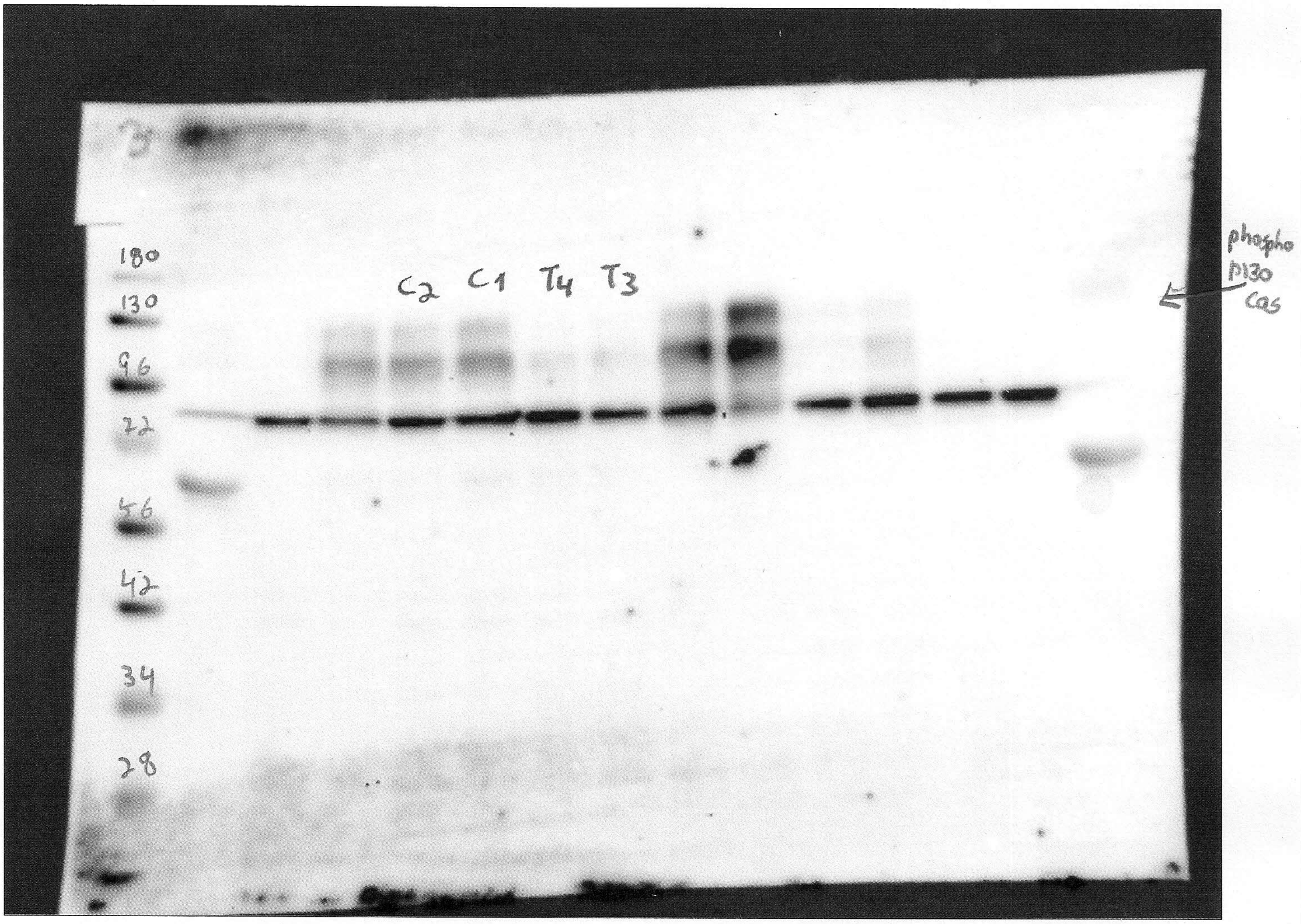

merge veredho 2015-08-17 14hr 22min_Exposure_6.0sec+veredho 2015-08-17 14hr 24min

Transfected cell lines

ES2

GAPDH 17.8.15 (phospho p130 cas 4.8.15)

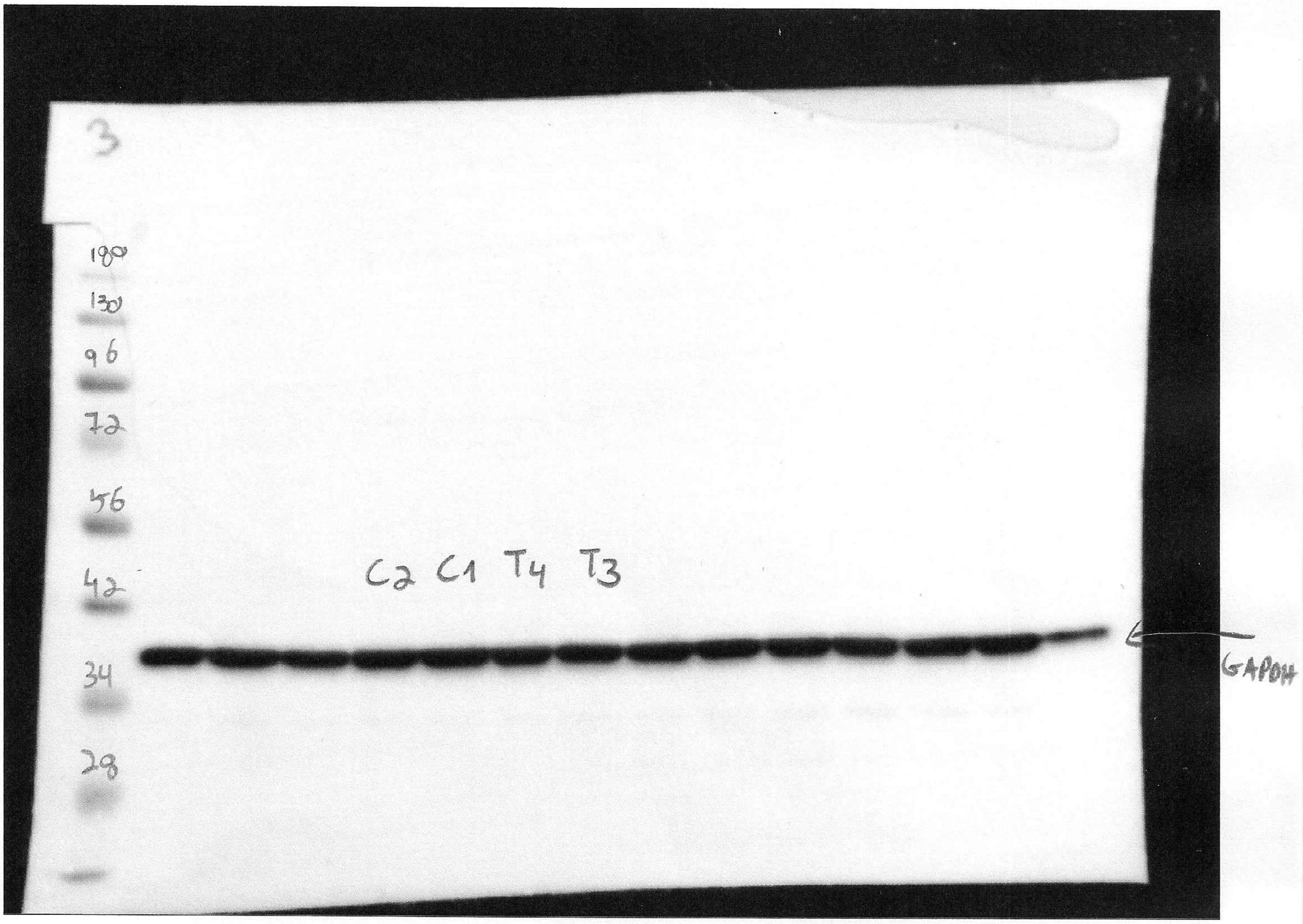

merge veredho 2015-08-04 11hr 20min_Exposure_13.4sec+veredho 2015-08-04 11hr 22min

Transfected cell lines
ES2
p130Cas 4.8.15

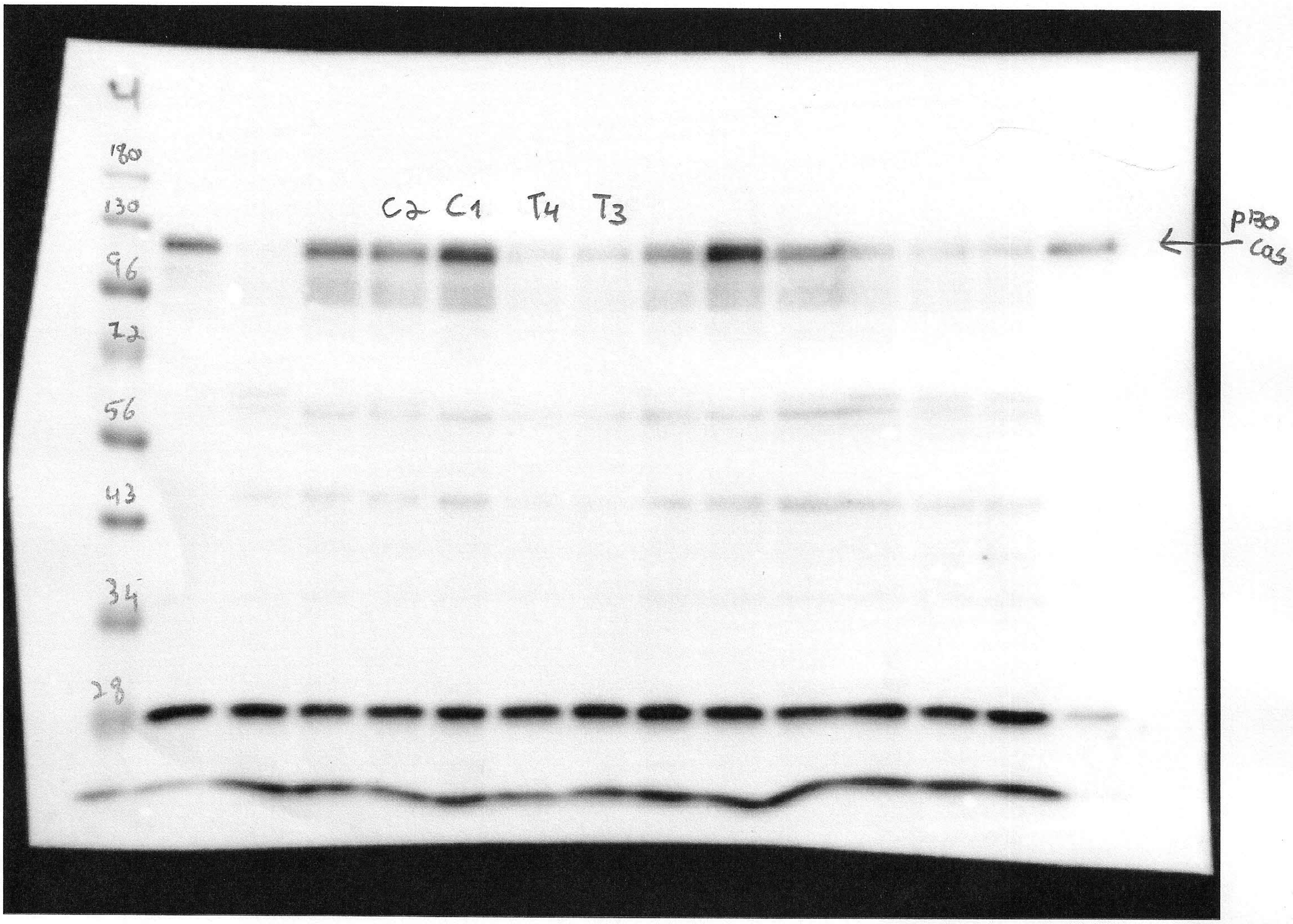

Transfected cell lines

ES2

GAPDH 17.8.15 (p130 Cas 4.8.15)

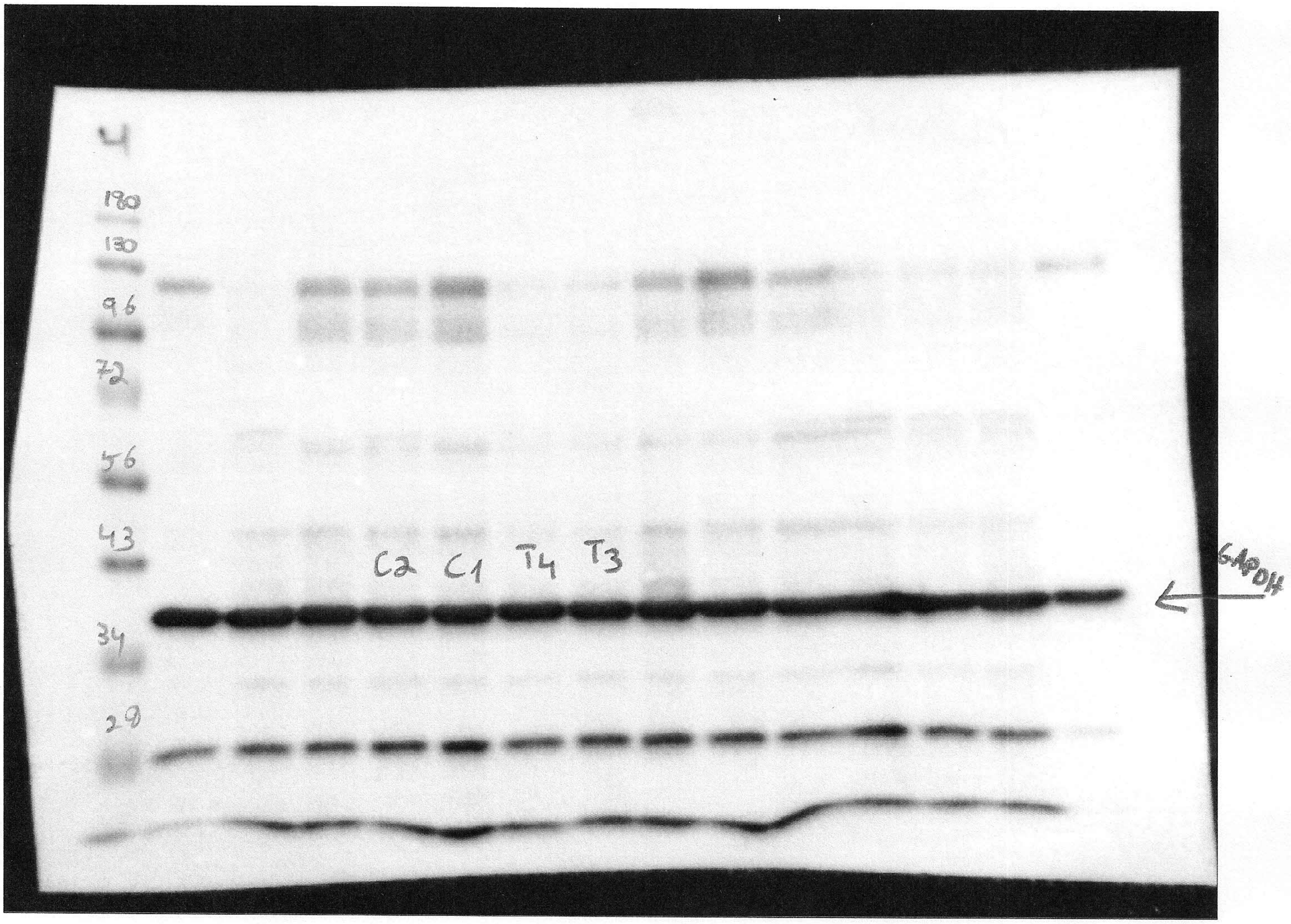

Transfected cell lines

OVCAR3

phospho ERM 6.8.15

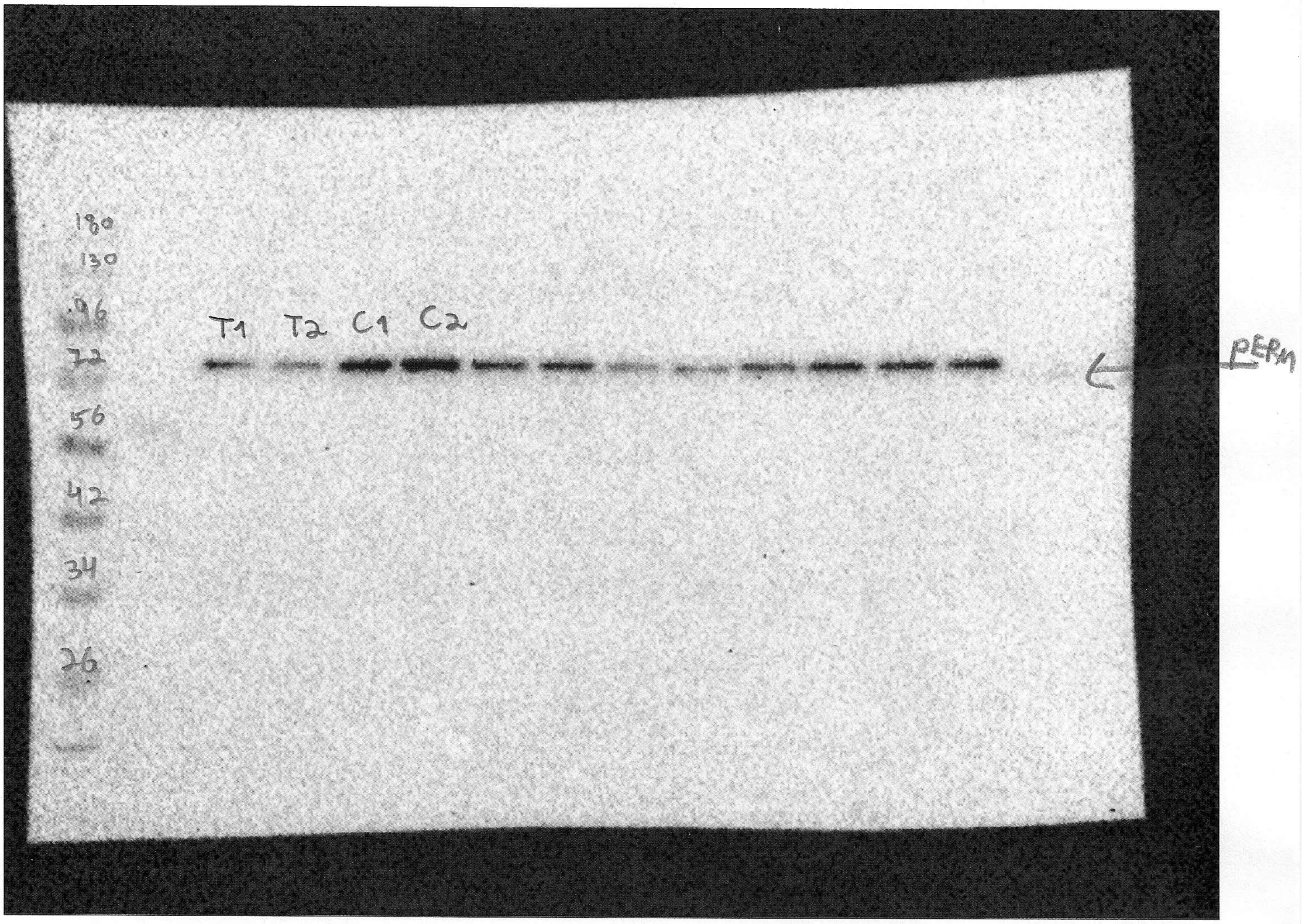

merge veredho 2015-08-17 14hr 02min_Exposure_7.0sec+veredho 2015-08-17 14hr 03min

Transfected cell lines
OVCAR3
GAPDH 17.8.15 (phospho ERM 6.8.15)

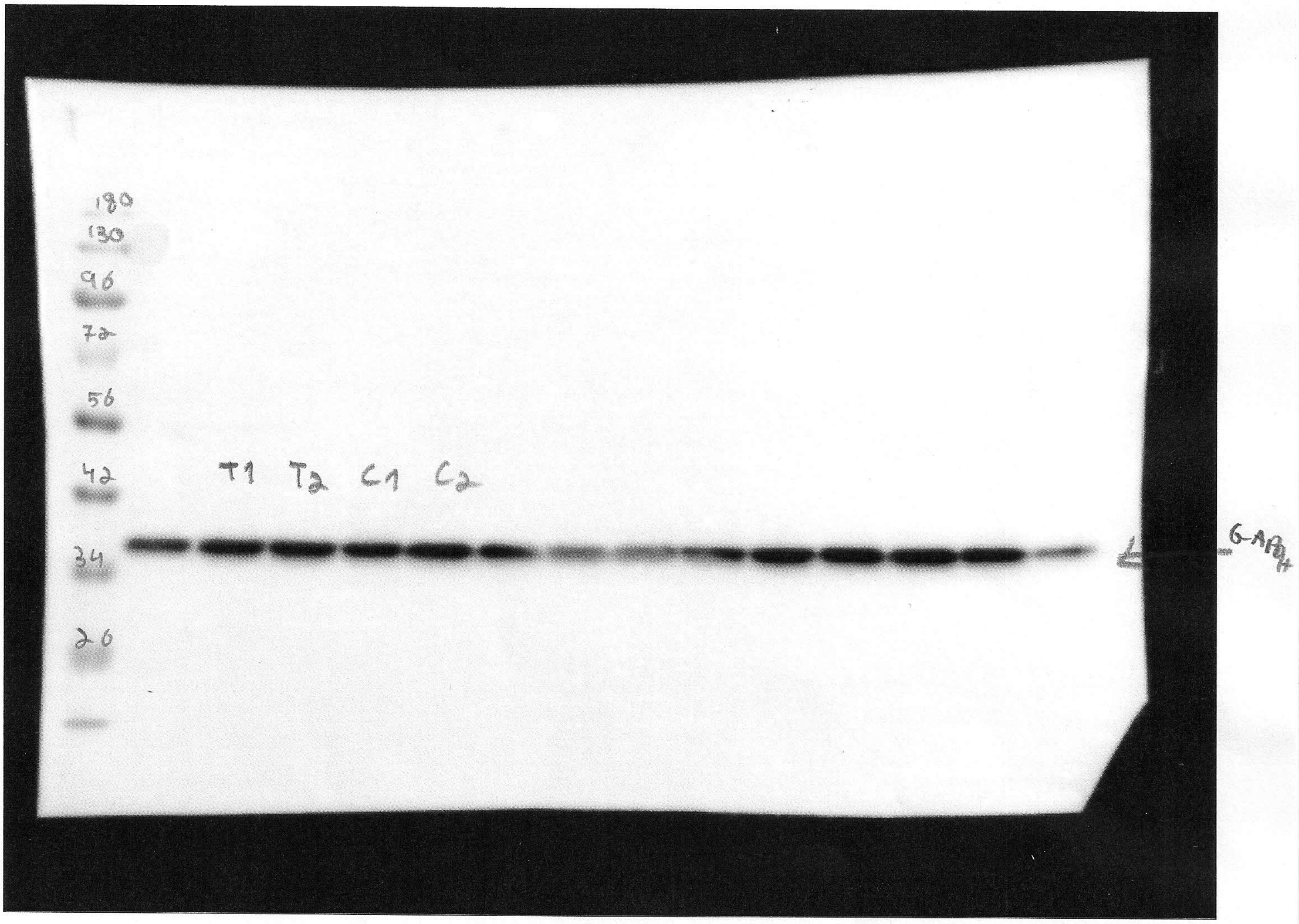

Transfected cell lines
OVCAR3
Ezrin 6.8.15

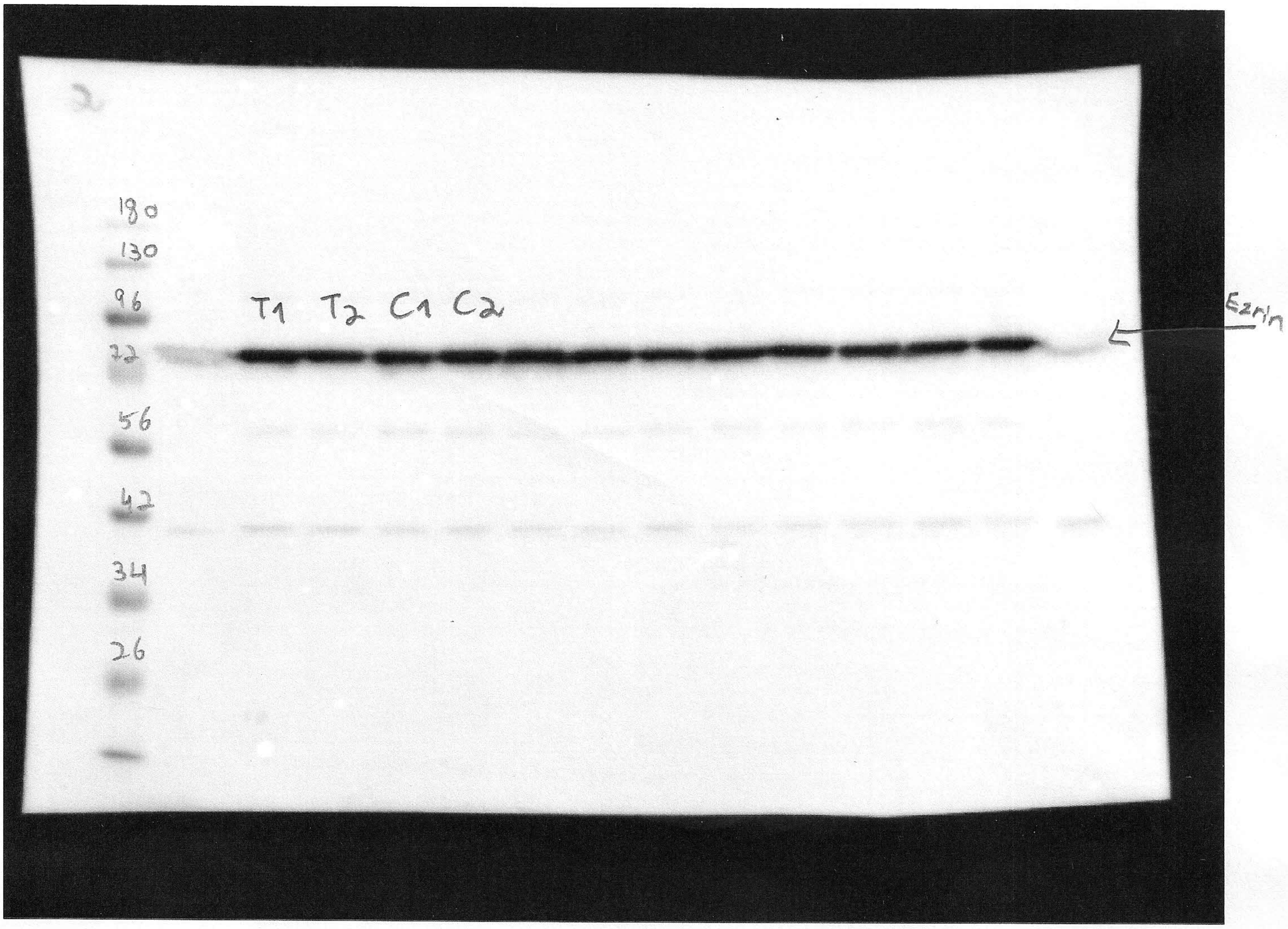

Transfected cell lines
OVCAR3
GAPDH 17.8.15 (ozrin 6.8.15)

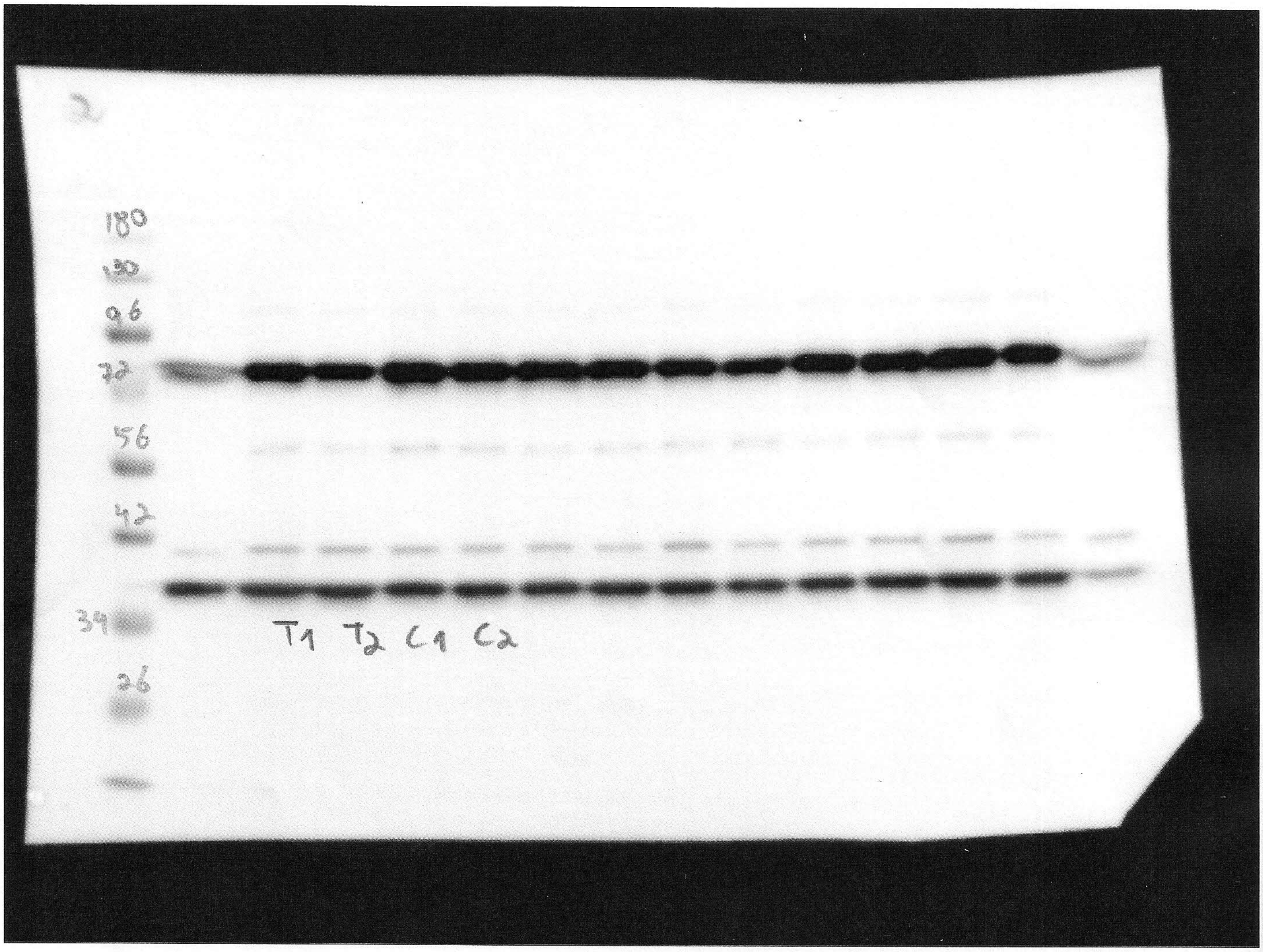

Transfected cell lines
OVCAR3
phospho-p130Cas 6.8.15

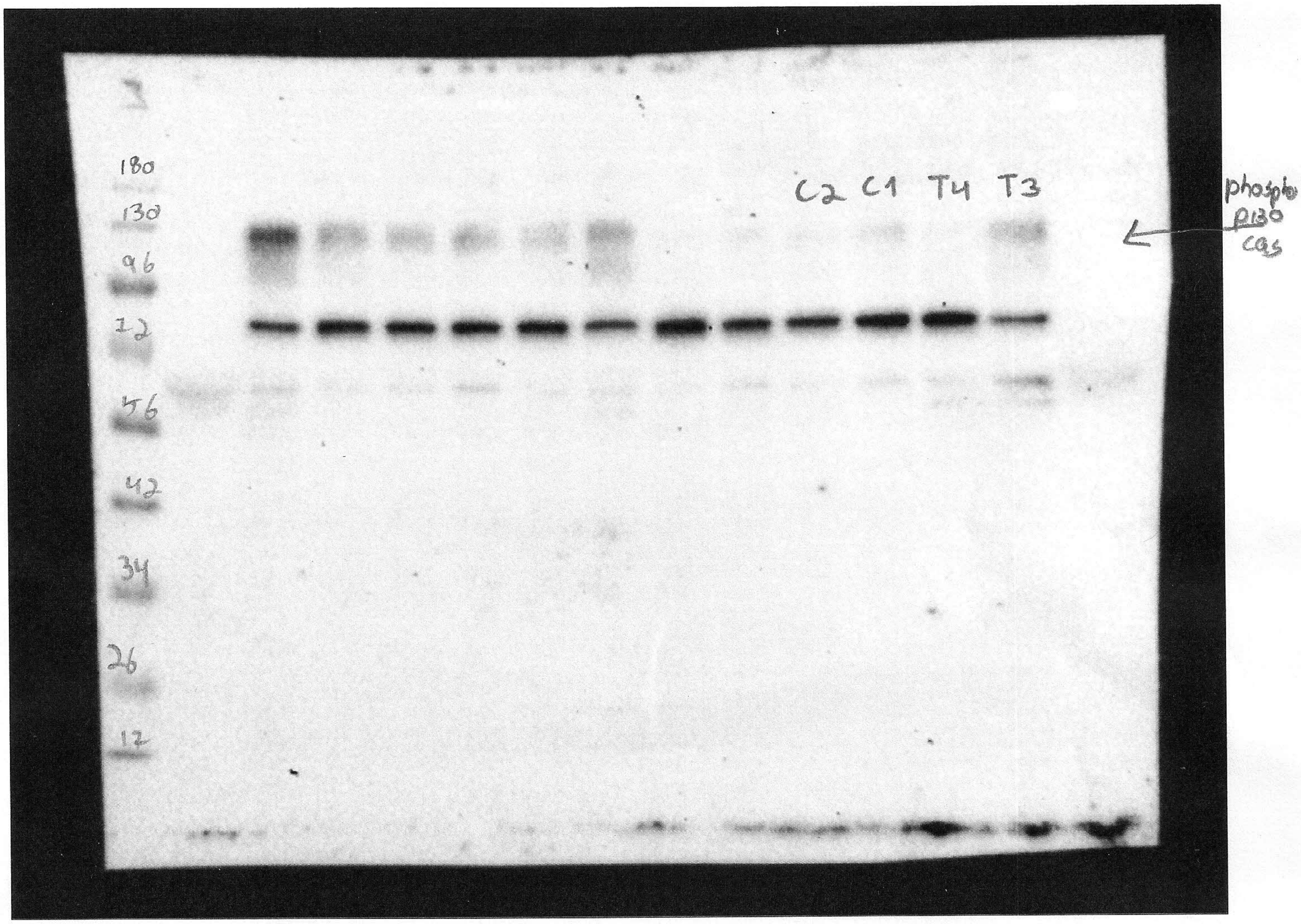

merge veredho 2015-08-17 14hr 09min_Exposure_30.0sec+veredho 2015-08-17 14hr 11min

Transfected cell lines
OVCAR3
GAPDH 17.8.15 (phospho p130cas 6.8.15)

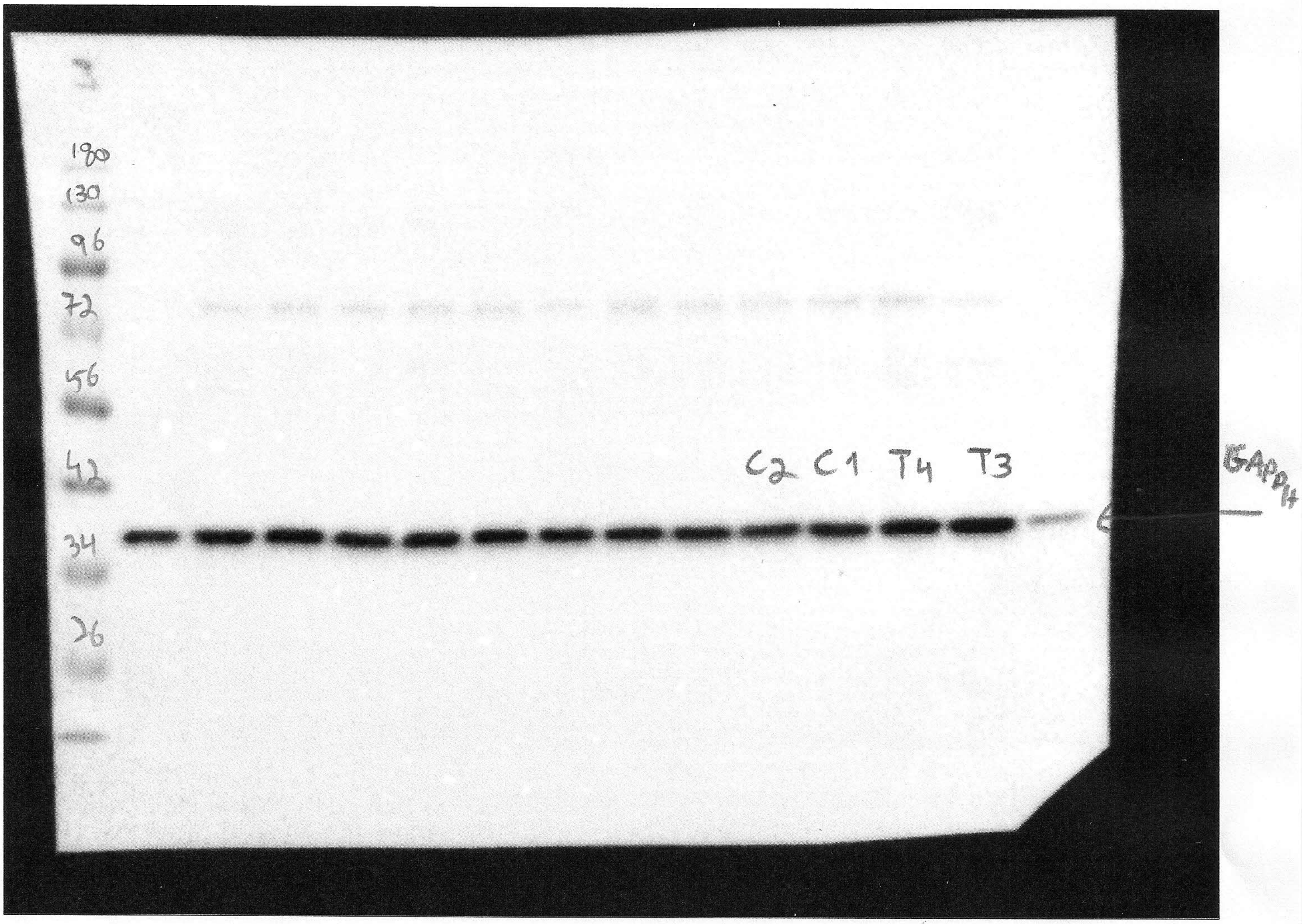

P:/labdata/reuvenr_lab_data/vered/vered 011014/western…ults/cell lines transfections/GAPDH/GAPDH OV3 3 170815
Printed: 30/06/2016 13:54:35

merge veredho 2015-08-06 11hr 55min_Exposure_19.6sec+veredho 2015-08-06 11hr 56min

Transfected cell lines
OVCAR 3
p130Cas 6.8.15

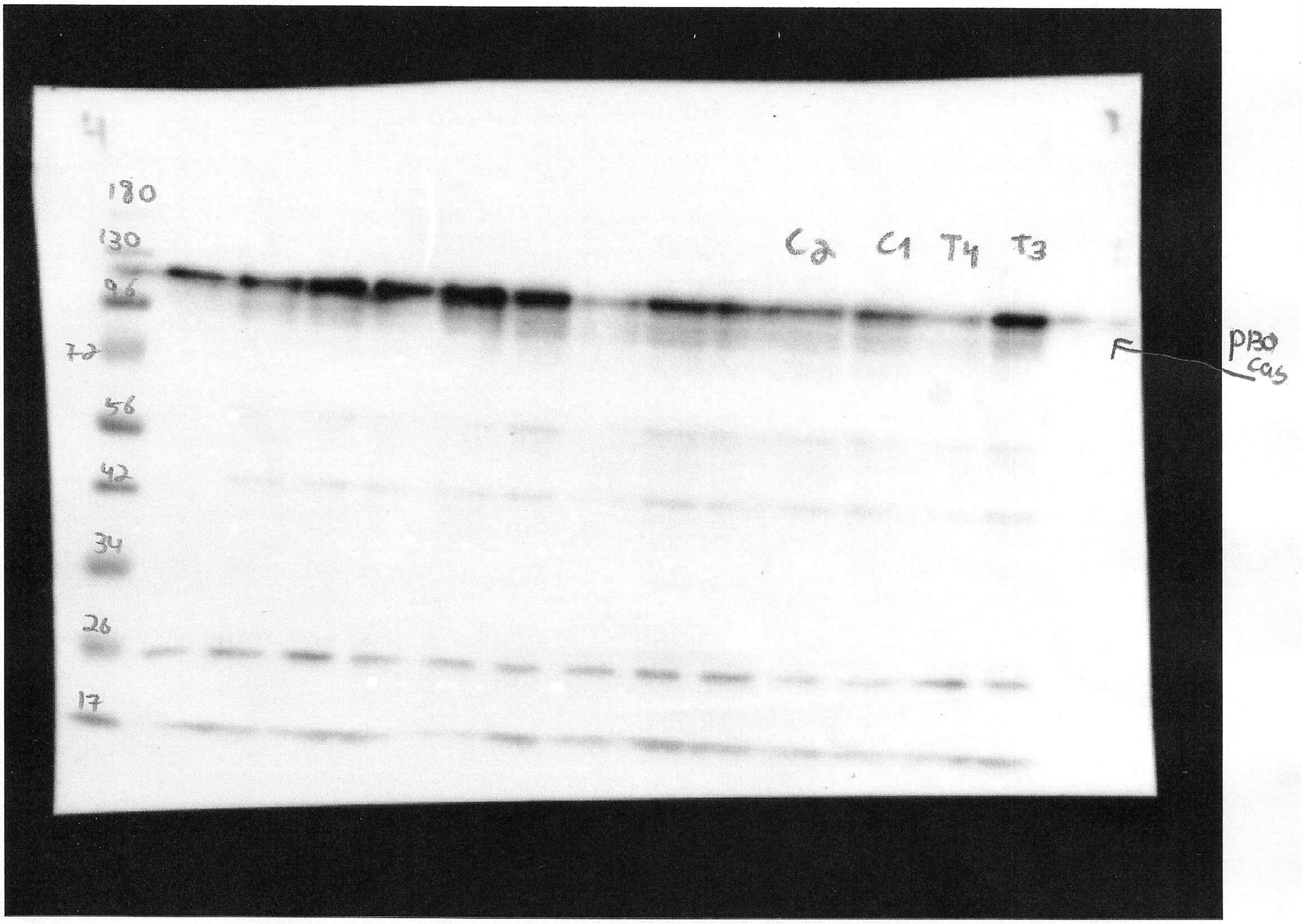

P:/labdata/reuvenr_lab_data/vered/vered 011014/western results/cell lines transfections/p130Cas/p130Cas 060815
Printed: 30/06/2016 12:27:24

Transfected cell lines
OVCAR3
GAPDH 17.8.15 (p130Cas 6.8.15)

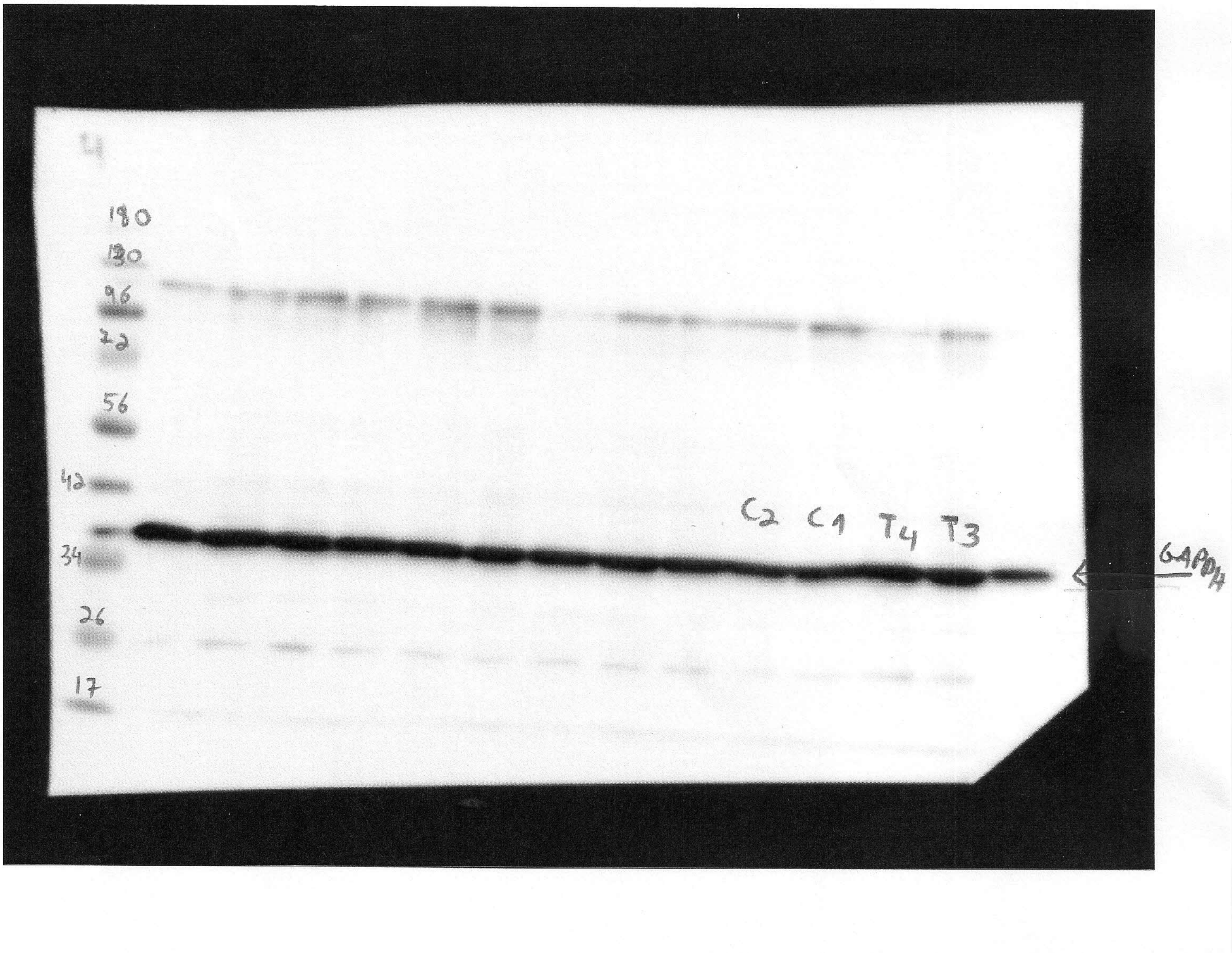

Supplement: S2 Fig — Expression of ezrin, p-ezrin, p130cas and p-p130cas in ES-2 and OVCAR3 cell lines. (PDF) [file pone.0162502.s002.pdf]
